# Machine Learning Methods for Fear Classification Based on Physiological Features

**DOI:** 10.3390/s21134519

**Published:** 2021-07-01

**Authors:** Livia Petrescu, Cătălin Petrescu, Ana Oprea, Oana Mitruț, Gabriela Moise, Alin Moldoveanu, Florica Moldoveanu

**Affiliations:** 1 Faculty of Biology, University of Bucharest, 050095 Bucharest, Romania; 2 Faculty of Automatic Control and Computers, University Politehnica of Bucharest, 060042 Bucharest, Romania; catalin.petrescu@upb.ro (C.P.); anaoprea98@yahoo.com (A.O.); oana.balan@cs.pub.ro (O.M.); alin.moldoveanu@cs.pub.ro (A.M.); florica.moldoveanu@cs.pub.ro (F.M.); 3 Faculty of Letters and Sciences, Petroleum-Gas University of Ploiesti, 100680 Ploiesti, Romania; gmoise@upg-ploiesti.ro

**Keywords:** emotion dimensions, emotion classification, fear classification, neural networks, machine learning

## Abstract

This paper focuses on the binary classification of the emotion of fear, based on the physiological data and subjective responses stored in the DEAP dataset. We performed a mapping between the discrete and dimensional emotional information considering the participants’ ratings and extracted a substantial set of 40 types of features from the physiological data, which represented the input to various machine learning algorithms—Decision Trees, k-Nearest Neighbors, Support Vector Machine and artificial networks—accompanied by dimensionality reduction, feature selection and the tuning of the most relevant hyperparameters, boosting classification accuracy. The methodology we approached included tackling different situations, such as resolving the problem of having an imbalanced dataset through data augmentation, reducing overfitting, computing various metrics in order to obtain the most reliable classification scores and applying the Local Interpretable Model-Agnostic Explanations method for interpretation and for explaining predictions in a human-understandable manner. The results show that fear can be predicted very well (accuracies ranging from 91.7% using Gradient Boosting Trees to 93.5% using dimensionality reduction and Support Vector Machine) by extracting the most relevant features from the physiological data and by searching for the best parameters which maximize the machine learning algorithms’ classification scores.

## 1. Introduction

As there is a broad interest in the field of affective computing and particularly, affect recognition, this study aims to explore fear classification based on extracted time-related, frequency-related and events-related features from a well-known dataset containing physiological recordings (electrodermal activity—EDA and heart rate variability—HRV) and self-reported ratings of valence, arousal and dominance. We extracted 40 types of features—33 related to EDA and 7 related to HRV. By combining the discrete and dimensional emotion models, we considered that fear is characterized by low valence, high arousal and low dominance. In our pursuit, we applied various machine learning algorithms—Decision Trees (DT), which are intuitive, transparent to inspection and easy to validate, k-Nearest Neighbors (kNN), Support Vector Machine (SVM) and artificial networks—and tackled various situations, such as dealing with an imbalanced dataset via data augmentation, preventing overfitting through cross-validation and by plotting learning curves, reducing dimensionality, selecting the most relevant features and tuning the appropriate hyperparameters in order to obtain the highest classification scores.

Particular attention was given to the data augmentation technique called Synthetic Minority Oversampling Technique (SMOTE), which generated new samples from the minority class, based on the closest examples in the feature space. Together with the undersampling of the majority class, this method helped to resolve the issue of working on an imbalanced dataset.

We applied the Local Interpretable Model-Agnostic Explanations (LIME) method for explaining the predictions, considering the features that increase the chances of obtaining the output 0 (no fear) or 1 (fear). Thus, we employed feature selection and feature importance scores, as well as other methods aimed at explaining the predictions of the neural networks—visual inspection of the learning curves, tuning the number of layers and number of neurons and adding dropout, which controls which features contribute most strongly to the neural network’s output.

Thus, we aimed to understand why a certain model behaves in a certain way and how it reaches a decision or a prediction, based on the input it is provided, the training method, feature selection and feature engineering. This is a highly necessary request in the medical field, where the end users need to trust that the artificial intelligence algorithms are making correct decisions.

We performed a global interpretation, by examining the models from a broader perspective, and tried to see which set of features maximized the classification scores. Additionally, we searched for rules that generalized to future real-world data outside the test set and tried to understand the models’ behavior in order to evaluate which training inputs mostly influenced a certain behavior.

Feature extraction presents the most relevant features from a biological point of view for accurately detecting the emotion of fear. Furthermore, our intuition for the reasons for which more complex neural network configurations do not reach good performances was encouraged by the fact that our data are almost linearly separable in a high dimensional space, as resulting from applying the SVM algorithm.

Two datasets were provided as input to the machine learning algorithms: the non-overlapping dataset with 120 features and the overlapping dataset, with 40 features.

The results show that all the tested algorithms provided statistically similar results, either with or without dimensionality reduction and feature selection. However, the highest performance (over 89%) for both datasets was achieved by the SVM and Gradient Boosting Trees (GBT) algorithms. For the non-overlapping dataset, by selecting the top 40 features using Recursive Feature Elimination Feature Selection and then by applying the SVM algorithm, we reached a performance score of 92.7%. For the overlapping dataset, by using all 40 features, the GBT algorithm had a 91.7% performance score. By selecting only the top 30 features either with XGBoost Feature Selection or Random Forest Classification Feature Selection and then applying the kNN algorithm, the performance dropped to 82.5%.

The significance of the study lies in approaching fear, an emotion sometimes considered rather a disadvantage, which in reality is very important, being the main emotion to which we owe survival. However, exacerbated fear, which is part of the psychopathological spectrum, prevents us from responding adaptively to life’s challenges.

The motivation of this study is given by the need to design a simple and efficient fear identification algorithm, which provides real-time information useful for guiding therapy sessions. The balance between the efficiency and simplicity of the algorithms will allow their implementation in a wearable device usable in psychological therapy (phobias, anxiety, depression). The article presents several aspects of novelty that cover current gaps in knowledge: (i) identifying and using a minimum number of features that carry a maximum amount of information, using both data analysis techniques and validating the selection based on their biological relevance; (ii) the automatic extraction of features using an in-house-developed MATLAB software that we intend to extend to a wearable device; and (iii) a fear identification approach based on emotional affective dimensions such as low valence, high arousal and low dominance using the DEAP (Dataset for Emotion Analysis using Physiological Signals) dataset for training the classifier.

The paper is structured as follows: Section 2 introduces fear, an adaptive emotional response; Section 3 presents the physiological data—RV and EDA; Section 4 comments on the most relevant studies published in recent years (particularly in the interval 2019–2021); Section 5 describes the framework and the protocol we followed; Section 6 presents the feature extraction methodology; Section 7 introduces the machine learning and deep learning methods that were used; Section 8 presents the results; Section 9 discusses on the results, provides a comparison with similar studies and sheds light on the study’s current limitations; and Section 10 summarizes with the conclusions and proposes future research directions.

## 2. Fear, an Adaptive Emotional Response

Emotions play an adaptive role that allows both humans and animals to act quickly and appropriately through actions that maximize the chances of survival and success. Emotions influence cognitive processes and behavior, being expressed through facial expressions, voice, behavioral responses and physiological reactions [1]. Understanding other people’s emotional displays gives us important information about how we must behave in response to a particular situation.

Fear is one of the fundamental human emotions, which occurs in response to an external threat that endangers the life or integrity of the being, which is real or perceived as real. Although it is a negative emotion and generally unpleasant, fear is useful because it plays an important role in our safety and integrity. For a fast and efficient discrimination of potentially harmful events, the amygdala, a collection of nuclei in the medial temporal lobe, is a central neural node both in predatory and social dominance contexts [2]. With connections to the brainstem, hypothalamus and cortical areas, the amygdala controls psychophysiological and behavioral responses that are commonly considered to be symptoms of fear and anxiety [3]. Fear is manifested through interdependent reactions of biochemical (hormonal), physical and behavioral nature. Physical, bodily, specific reactions can be: sweating, trembling, an increased heart rate, shortness of breath or difficulty in breathing, muscle tension or increased adrenaline levels.

There are two distinct models for classifying emotions: *the categorical (discrete)* and the *dimensional model.* In the categorical model, there are six basic, distinct and universal emotions: happiness, anger, sadness, surprise, disgust and fear [4,5,6]. Because complex affective states cannot be expressed by a single label, the researchers used the dimensional model, with two fundamental emotional spaces: valence and arousal (the circumplex model) [7]. Valence describes the affective quality of the emotion, being a measure of the pleasantness that an event generates, specifying how positive (pleasant) or negative (unpleasant) it is. Arousal characterizes the intensity of the physical and cognitive stimulation and ranges from low arousal (passive) to high arousal (active) [8]. In the classical circumplex model of affect, the emotions of fear and anger are indistinguishable, because they both lie in the same quadrant of low valence and high arousal. Researchers have found dominance (defined as the capacity of being in control of one’s emotions) as a third dimension, very important to represent the emotional space in the Pleasure–Arousal–Dominance (PAD) model. Dominance represents the “fight or escape” reaction to stimuli. In that new dimensional model, fear was described by negative valence, high arousal and low dominance, while anger is defined by negative valence, high arousal and high dominance [9,10,11].

Although it has ensured the survival and evolution of the human species over the centuries, prolonged, irrational fear is harmful both physically and emotionally, affecting the quality of life, daily activities and communication. This is why the early and efficient identification of disorders such as those in the anxiety spectrum is a direction with many approaches. The field of affective computing uses technology to enhance emotion recognition and aims to develop applications that automatically adapt to unexpected emotional changes. Multimodal emotion recognition systems have higher classification accuracies, depending on the number of emotions, extracted features, classification methods and the quality of the physiological dataset [12]. Biosensors can detect emotions by monitoring physiological signals, which are the response to the activity of the Autonomic Nervous System (ANS)—HRV, EDA, temperature and respiration. These kinds of systems are wearable and unobtrusive [1], being included in the category “affective wearables” [13].

## 3. Physiological Data

### 3.1. Heart Rate Variability

A healthy heart does not have the rhythm of a metronome. On the contrary, it is characterized by oscillations with a complex and constantly changing pattern, as a result of the rapid adaptation of the cardiovascular system to sudden physical and psychological challenges to homeostasis [14]. These oscillations reflect the dynamic intervention of the ANS in the regulation of the internal organs’ activity. HRV is considered one of the main non-invasive methods for assessing the function of ANS, the heart being equally innervated by both sympathetic and parasympathetic pathways. The sympathetic nervous system (SNS) plays a positive role, increasing heart rate, atrioventricular conductivity and cardiac contractility, while the parasympathetic nervous system (PNS) decreases these parameters to ensure the adequate rest and energy reserves of the heart. Accordingly, HRV is a dynamic index of interdependence between the ANS pathways, being an indicator of adaptation to environmental and psychological challenges [15]. ANS is further regulated by the central autonomic network (CAN) which consists of cortical (medial prefrontal and insular cortices), limbic (anterior cingulate cortex, hypothalamus, the central nucleus of the amygdala, the bed nucleus of the stria terminalis) and brainstem regions [16,17]. HRV measures the beat-to-beat temporal changes of heart rate, being usually represented by the variation in the RR intervals collected from the electrocardiogram (ECG) data. It can be measured from both ECG and photoplethysmography (PPG) signals, which correlate in the proportion of 88% [18]. The PPG sensor uses a light-emitting diode to record the waveform of the pulse and can be placed anywhere on the body, due to its high sensitivity in detecting the pulse waves even from the smallest capillaries [12]. PPG changes quickly with emotional reactions, but has the disadvantage of being vulnerable to vibrations and movement artifacts [19]. Heart rate increases with arousal and decreases in the presence of unpleasant stimuli, being a reflective index of emotional regulation [16].

### 3.2. Electrodermal Activity

EDA, also known as the Galvanic Skin Response (GSR), is a measure of neuro-physical arousal, estimating the changes in skin conductance as a response to the ANS activity. EDA is a valuable tool in psycho-behavioral studies because it is a correct indicator of the SNS response, being generally difficult or impossible to influence or control the production of eccrine sweat from the skin as a response to emotional stimuli. Additionally, the non-invasive nature of this technique makes it preferable to other approaches.

The EDA mechanism is highly modulated within the limbic system via the hypothalamus and the thermoregulatory pathways, and to a lesser degree by the premotor cortex and the basal ganglia [20].

For the evaluation of EDA, the signal that characterizes the evolution in time of the skin conductance is measured. It is hereinafter referred to as the GSR signal, for consistency with the name used in the DEAP database. GSR signals can be decomposed into two quantitative components, in response to specific tasks:

- Skin Conductance Level (SCL)—the tonic component, a measure of continuous, slowly changing background characteristics (mean value 2–20 μS);
- Skin Conductance Response (SCR)—the phasic component, with rapid changes associated with specific and identifiable stimuli, as a result of momentary SNS activation (mean value 0.1–1.3 μS) [21].

The spontaneous fluctuations in EDA responses can also be recorded, without an association with a specific stimulus (they occur more than 5 s after a stimulus). An increased frequency of Non-Specific SCR (NS-SCR) and a high level of SCL are relevant markers of stress and anxiety [22].

## 4. Related Work

Emotion classification is an emerging field which has drawn the attention of researchers from various domains. The development of physiological monitoring devices, signal-processing algorithms and artificial intelligence methods, including fuzzy optimization [23], has recently enabled the expansion of automatic emotion recognition systems.

Using as stimuli for emotions the dataset called testImages_artphoto from [24], a new approach for emotion recognition with high arousal was presented in [25]. Four emotions were considered: sadness, contentment, anger and fear. The process was iterative and user centered. Each iteration consisted of five steps: data acquisition, data preprocessing, emotion classification, user emotions collection using the Self-Assessment Manikins (SAM) questionnaires [9] and evaluation. The data acquired were heart rate, movement and audio. The data preprocessing consisted of synchronization and data normalization. For emotion classification five models were used: Multilayer Perceptron (MLP), Convolutional Neural Network (CNN), Long–Short Term Memory, Bidirectional Long–Short Term Memory and DT. K-fold cross-validation with k = 5 was performed to assess the models’ prediction rates. Accuracy and Receiver Operating Characteristic (ROC) were used to measure the performance of the five models. The best results were obtained with DT (the final accuracy was 91.47%) and the worst results with CNN (85.05% accuracy).

Highlighting the importance of the data preprocessing phase to improve the performance of the machine learning models, a comparative analysis between various machine learning techniques, such as SVM, kNN, Linear Discriminant Analysis (LDA), Logistic Regression (LR) and DT for emotion recognition on the DEAP dataset [26], was performed in [27]. In order to understand the data and to investigate the most appropriate predictive models, the authors considered Exploratory Data Analysis as the first stage in the process of automatic emotion recognition. Valence, arousal, dominance and liking were independently classified using the classical ML algorithms. The metrics for performance (accuracy, precision, recall, F1 score) showed that the algorithms were somehow close in performance. Regarding the accuracy, the best algorithm was kNN, followed by SVM, DT, LR and LDA [27].

In the investigation of [28], a new approach was proposed for emotion identification. The authors considered four emotional states defined by two dimensions (high valence–high arousal (HVHA), high valence–low arousal (HVLA), low valence–low arousal (LVLA), low valence–high arousal (LVHA)) to be identified based on the GSR, PPG, respiration and electromyography (EMG) signals stored in the DEAP dataset. The new approach consisted of the following steps: four nonlinear features were extracted (approximate entropy—ApEn, sample entropy—SaEn, fuzzy entropy—FuEn and wavelet packet entropy—WpEn), then the extracted features were fused, and a team-collaboration identification strategy was employed to identify the emotional state. The team-collaboration strategy was a fusion of three ML techniques: SVM, DT and Extreme Learning Machine (ELM). The obtained average accuracy was 76.46%.

In [26], only the peripheral physiological signals from DEAP were analyzed. Eight machine learning techniques (SVM, kNN, Random Forest (RF), DT, LR, Gaussian Naïve Bayes (GNB), LDA and MLP classifier) for emotion classification were compared, using both standard and non-linear extracted features. Three combinations of data were investigated: raw peripheral physiological signals, a non-linear features set and a peripheral features fusion set. Principal Component Analysis (PCA) was used to reduce the dimensionality of the data. The performance of ML models was measured by accuracy and F1 score, which for valence classification varied between 61.25 and 64.92%, and 74.39 and 77.20%, respectively. For arousal classification, the accuracy was between 61.56 and 63.8% and F1 score between 75.57 and 77.57%. The conclusions drawn by the authors were that the emotions can be recognized using peripheral physiological signals and overall SVM, LR and LDA outperform the KNN, DT, GNB, RF and MLP classifiers.

In [1], 37 users watched emotion-eliciting videos with a duration of 2 minutes and 40 s while PPG and GSR signals were recorded through an instrumented glove. A set of 27 features in time and frequency domain were extracted. Three emotional states were classified—amusement (positive valence and positive arousal), sadness (negative valence and negative arousal) and neutral, located in the center of the circumplex model of affect. The best model was obtained with RF recursive feature elimination for feature selection and SVM for the classification of amusement (96%) and sadness (91%). The model was validated on the DEAP dataset and the algorithm that maximized the F1 score was bagging tree (81%). For amusement and sadness, the PPG features were not significant because these emotions have similar values of arousal. Arousal is influenced by heart rate and heart rate is extracted from the PPG signal. Thus, amusement and sadness can be detected only from GSR features.

The DEAP database has been used for classifying valence and arousal based on the PPG signal and the normal-to-normal (NN) interval of HRV in time domain and frequency domain. The best classification results were obtained by two convolutional neural networks with 10 selected statistical features (three features in time domain and seven features in frequency domain)—82.1% for valence and 80.9% for arousal. The Pearson correlation coefficient showed a higher correlation between valence and the statistical features than between arousal and the statistical features [19]. In [29], respiration and HRV signals were collected from 53 participants who watched video clips eliciting six basic emotions (happiness, fear, surprise, anger, sadness and disgust). The multi-signal (respiration rate and HRV in both time and frequency domain) classification accuracy using a CNN model was on average 94%, and for the emotion of fear, in particular, 95.83%. The authors also concluded that it is efficient to classify emotions with multi-signals, where both domains of HRV and respiration rate should be used. CNNs provide very good results, but at the cost of high computing requirements.

Using only HRV features for emotion detection (sadness, anger, fear, happiness, relaxation), the highest classification accuracy (56.9%) was achieved by the SVM algorithm [30]. EDA features contributed to a classification accuracy of 64.32% for four levels of arousal.

The study described in [31] presents a binary fear recognition system (fear was considered as a low valence and high arousal emotion) based on 20 PPG and GSR features extracted from the DEAP dataset, using the subject-independent modality. The best results—concerning the values of accuracy, specificity and sensitivity—were obtained by applying the SVM algorithm, a dimensionality reduction technique based on Fisher’s Criterion and the SMOTE method for dealing with the class imbalance problem (there were 979 negative observations and 301 positive observations). The classification results were: accuracy—62.35%, sensitivity—62.27% and specificity—62.41%.

A novel study from 2021 [32] developed a specialized fear recognition system –using only women participants. It relied on binary classification based on a combination of linear (temporal and frequency) and non-linear features extracted from the GSR, ECG and skin temperature signals stored in the MAHNOB dataset [33]. The authors proposed a mapping of the emotion of fear onto the discrete and dimensional models. Thus, fear was characterized by low valence, high arousal and low dominance. The best accuracy was obtained by the Ensemble Classifier (ENS)—96.33% for the subject-dependent modality and 76.67% for the subject-independent modality using the Leave One Subject Out testing approach. The limitations of the study lie in the fact that only the data from 12 subjects (the women from the MAHNOB dataset) were used in the classification process.

In research published in 2019, we developed a binary fear recognition system in which we mapped the discrete and dimensional emotion models and considered fear as an emotion with low valence, high arousal and low dominance. The study is presented in [34]. The system was validated on the DEAP dataset as well, but it used all the biophysical types of data—heart rate, galvanic skin response, respiration, EMG, temperature and in addition, electroencephalogram. In that study, we did not perform data segmentation, windowing or feature extraction, but instead we used all the raw signals available in DEAP, without any pre-processing. The same approach was applied for the other emotions from the discrete model—anger, joy, surprise, disgust and sadness in our study presented in [35].

In this paper, we considered only the heart rate and galvanic skin response recordings from the DEAP dataset. A total of 33 features in the time-, frequency- and events-related domains were extracted from the GSR signal and another seven from the HR channel. We considered only GSR and HR because they can be easily recorded by lightweight wearable devices and have a larger usability for experimental purposes. In contrast with the research carried out in [34,35], in this one we performed denoising, data segmentation and windowing with and without overlapping, we applied low computational complexity binary classification algorithms with and without feature selection and dimensionality reduction, grid searched for the most relevant hyperparameters in order to obtain the best classification models and calculated various metrics, including the ROC AUC score, known to be a very reliable indicator of classification accuracy for imbalanced datasets.

## 5. Research Design Framework

**Figure 1:**
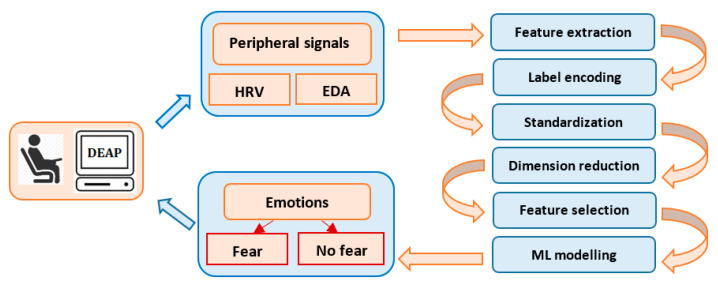
Data processing flow.

Our interest is to develop a simple, machine learning model to classify emotions using the peripheral signals from the DEAP dataset [36]. DEAP is a popular and consistent emotion database used for research purposes in a multitude of studies, having pre-processed data in both Matlab and Python formats, a comprehensive documentation and an appropriate description. The study focuses on the recognition and binary classification of the emotion of fear, based on the physiological data and subjective responses stored in the DEAP dataset. Automatic fear recognition and classification is an important approach, because it contributes to the development of medical and behavioral applications, such as anxiety, depression, phobias or post-traumatic stress disorder. Figure 1 describes the data-processing flow, starting with the extraction of the subject’s physiological characteristics from EDA and HRV. Fear classification is performed using different machine and deep learning algorithms and feature selection techniques.

## 6. DEAP Feature Extraction

### 6.1. DEAP Description

The DEAP database [36] consists of a set of electrophysiological recordings from 32 participants who watched 40 extracts of music videos. After watching each video extract, the participants rated the video in terms of arousal, valence, like/dislike, dominance and familiarity using a continuous nine-point scale. Electrophysiological data were recorded in 32 files using the BioSemi format containing the following signals:

- Electroencephalography (EEG) signals recorded from 32 channels;
- Electrooculography (EOG) signals recorded from eight channels;
- GSR signals corresponding to the EDA;
- Respiration signal recorded from a respiration belt;
- PPG signal measured on the left thumb, corresponding to HRV;
- Temperature signal measured on the left little finger;
- Status signal containing the markers sent from the stimuli presentation computer.

All signals were continuously recorded during each trial session, using a sampling rate of 512 samples/s.

### 6.2. DEAP Features Extraction Protocol

**Figure 2:**
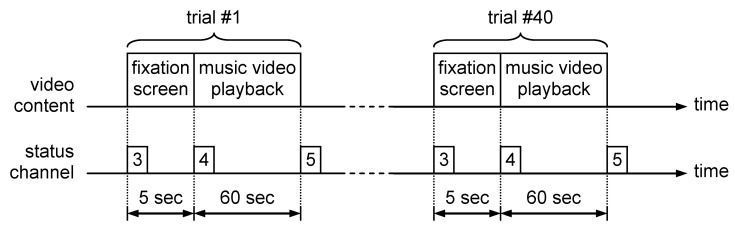
DEAP trial session structure.

The biophysical signals selected for *valence*, *arousal* and *dominance* evaluation were GSR and HRV. The reason for choosing these signals was the simplicity of setting the measurement process, that requires a small number of electrodes that are easy to be placed. In order to process the electrophysiological data contained in DEAP database, the content of each trial session file was read using a software developed by the authors. This software converts the BioSemi file format into a .csv file that contains three columns of data corresponding to skin conductance, heart rate and status signal. The .csv files were opened using a MATLAB processing script with the **csvread()** function. The next step was to extract the signal segments corresponding to each of the 40 trials present in the trial session file. This segmentation process was based on the values contained in the status signal, as presented in Figure 2.

Each trial that corresponds to watching a 60 s extract of music video was preceded by a 5 s interval in which a fixation screen was displayed. Because the fixation screen was emotionally neutral, this interval allowed us to record the baseline value for all electrophysiological signals. In parallel with signal segment extraction, the video ratings collected from the trials were read from the “participant_ratings.xls” file provided in the DEAP database. Each video was characterized by a continuous numerical value between 1 and 9, assessed for arousal, valence, like/dislike, dominance and familiarity. The fear perceived by the participant while watching the clip was evaluated on a binary scale (0—absence of fear/1—presence of fear) based on the following rule [10]:

- fear = 1 if valence ≤ 5 AND arousal > 5 AND dominance ≤ 5;
- fear = 0 in rest.

**Figure 3:**
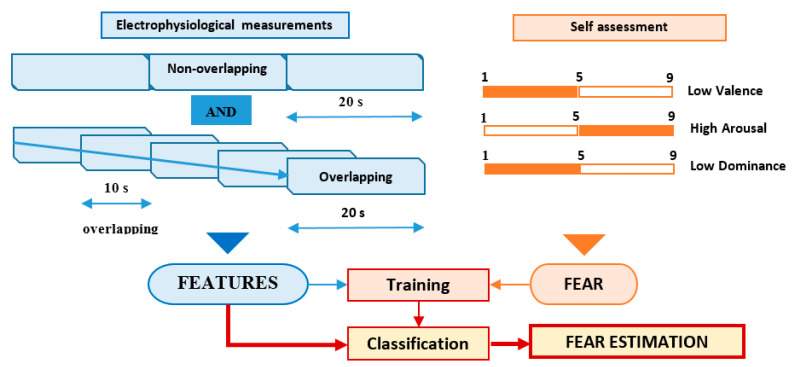
Fear classification process.

**Figure 4:**
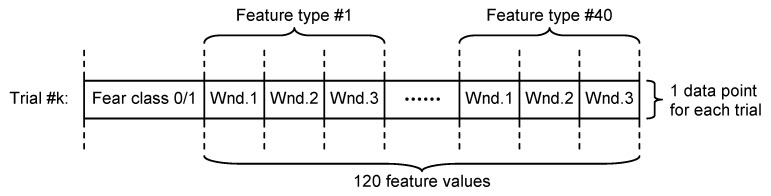
Dataset structure for non-overlapping approach.

**Figure 5:**
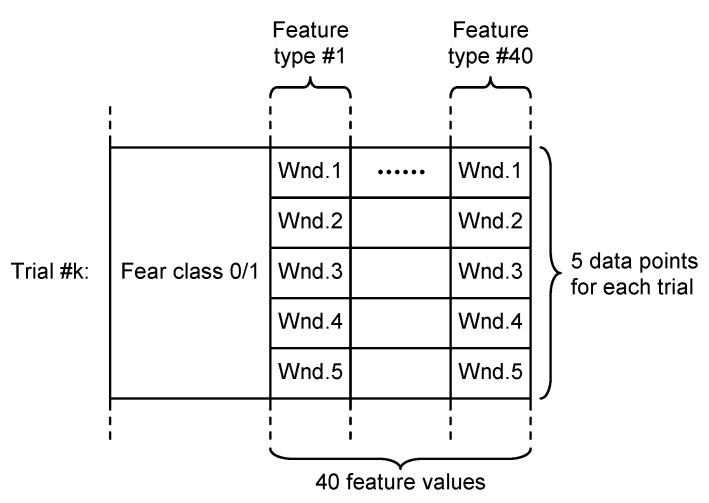
Dataset structure for overlapping approach.

The features of the electrophysiological signals (40 types of time-related, frequency-related and event-related features) were associated with each trial using two different approaches, according to Figure 3:

- Non-overlapping approach—each of the 60 s trials was segmented in three non-overlapping windows of 20 s length each. All signal features were evaluated in each window, the three values obtained for each feature being considered as independent. For this approach, for each trial, we evaluated a number of 120 features (3 segments × 40 features on each segment), as can be seen in Figure 4.

- Overlapping approach—each trial was segmented in five windows of 20 s length and 10 s overlap. Thus, five sets of 40 feature values were obtained for each trial, all of them being associated with the fear evaluated for that trial acting as five different data points, as can be seen in Figure 5.

The data from three subjects (number 23, 26 and 29) was discarded from the analysis because it contained invalid information that could not be processed.

### 6.3. Electrodermal Activity Features Extraction

In order to obtain the skin-conductance-related features, the measured GSR signal was decomposed into the SCL and SCR components using the Continuous Decomposition Analysis (CDA) algorithm [37] implemented in the Ledalab software. This algorithm also detects individual and non-superimposed SCRs and evaluates their amplitudes.

Before applying the CDA algorithm, the GSR signal was filtered and sub-sampled to 16 samples/s. This filter eliminated all power-grid-induced noises and also acted as an anti-alias filter prior to sub-sampling. A low-pass Finite Impulse Response (FIR) was selected due to the following reasons:

- FIR filters have linear phase characteristics that help to preserve the waveshape of the signal;
- This class of filters is more suitable for fixed-point arithmetic implementation. This aspect is very important, especially for building the portable fear level estimation device that will use a low power microcontroller without floating point computing capabilities.

The filter was designed using the window method (function **fir1()** from MATLAB Signal Processing Toolbox™). Design parameters were: cutoff frequency: 5 Hz, filter order: 512 and Hamming window type.

There were extracted 33 features including time- and frequency-related parameters.

*Time-related features* were directly computed from the signal samples corresponding to each time window.

**GSR_mav, SCL_mav** and **SCR_mav** represent the mean absolute values of GSR, SCL and SCR signals over the evaluation window:

(1) $$X\_mav=\frac{1}{N}\overset{N-1}{\underset{K=0}{\sum }}\left|X_{k}\right|\;,$$

where: X represents the analyzed signal X∈{GSR,SCL,SCR} and $X_{K}$ are their samples; N represents the number of signal samples included in the evaluation window.

**GSR_mar**, **SCL_mar** and **SCR_mar** represent the ratio between the mean absolute values of GSR, SCL and SCR signals over the evaluation window and over the baseline recording interval:

(2) $$X\_mar=\frac{X\_mav}{Xb\_mav},\;\;Xb\_mav=\frac{1}{M}\overset{M-1}{\underset{K=0}{\sum }}\left|Xb_{k}\right|,$$

where: $Xb_{k}$ represents the signal samples corresponding to the baseline recording interval; M represents the number of signal samples included in the baseline recording interval.

**GSR_std, SCL_std** and **SCR_std** represent the standard deviation of the signal samples over the evaluation window:

(3) $$X\_std=\sqrt{\frac{1}{N-1}\overset{N-1}{\underset{K=0}{\sum }}{\left(X_{K}-X\_mav\right)}^{2}},$$

**GSR_str**, **SCL_str** and **SCR_str** represent the ratio between the standard deviation of the signal samples over the evaluation window and over the baseline recording interval

(4) $$X_{str}=\frac{X_{std}}{Xb_{std}},\;\;\;\;Xb\_std=\sqrt{\frac{1}{M-1}\overset{M-1}{\underset{K=0}{\sum }}{\left(Xb_{K}-Xb\_mav\right)}^{2},}$$

**GSR_wl, SCL_wl and SCR_wl** represent the Waveform Length of the signals [38]:

(5) $$X\_wl=\overset{N-1}{\underset{K=1}{\sum }}{\left(∆X_{K}\right)}^{2},\;\;∆X_{K}=X_{K}-X_{K-1},$$

**GSR_ssc, SCL_ssc** and **SCR_ssc** represent the Slope Sign Changes of the signal [38]:

(6) $$X\_ssc=\overset{N-2}{\underset{K=1}{\sum }}f\left(X_{K-1},\;X_{K},\;X_{K+1}\right),$$

where:

$$f\left(X_{K-1},X_{K},X_{K+1}\right)=\{\;\begin{array}{c} 1\;if\;sign\left(X_{K}-X_{K-1}\right)*sign\left(X_{K+1}-X_{K}\right)<0\;and\\ \;\left|X_{K}-X_{K-1}\right|\geq \varepsilon \;and\\ \;\left|X_{K-1}-X_{K}\right|\geq \varepsilon \\ 0\;otherwise\; \end{array}$$

and the slope threshold ε=0.001 μS.

**GSR_wamp, SCL_wamp** and **SCR_wamp** represent the Willison Amplitudes of the signals [38]:

(7) $$X\_ssc=\overset{N-1}{\underset{K=1}{\sum }}f\left(X_{K-1},X_{K}\right),$$

where

$$f\left(X_{K-1},X_{K}\right)=\{\begin{array}{c} 1\;if\;\left|X_{K}-X_{K-1}\right|\geq \varepsilon_{W}\;\\ 0\;otherwise\; \end{array}$$

and the Willison threshold ε=0.5 μS.

*Frequency-related features* were computed from the power spectrum density (PSD) of the electrodermal signals corresponding to each time window. PSD was evaluated for a set of frequencies $\left\{f_{K=0\ldots P-1}\right\}$ between 0 and 8 Hz with a 0.01 Hz step.

**GSR_fmd, SCL_fmd** and **SCR_fmd** represent the Median Frequencies of the power spectrum [39]. It is the frequency $f_{Q}$ that divides the signal spectrum in two sections which have the same energy. The frequency index Q is obtained in order to minimize the following value:

(8) $$\left|\overset{Q-1}{\underset{K=0}{\sum }}P_{K}-\overset{P-1}{\underset{K=Q}{\sum }}P_{K}\right|$$

where $P_{K}$ represents the *K*th line of the PSD.

**GSR_fmn, SCL_fmn** and **SCR_fmn** represent the Mean Frequencies of the power spectrum [39]:

(9) $$X\_fmn=\frac{\sum_{K=0}^{P-1}f_{K}P_{K}}{\sum_{K=0}^{P-1}P_{K}},$$

**GSR_hlr, SCL_hlr** and **SCR_hlr** represent the ratio between the signal energy in the [0.2 Hz, 2 Hz] and [0.01 Hz, 0.2 Hz] frequency intervals [39]:

(10) $$X_{hlr}=\frac{\sum_{K=I_{b}}^{I_{C}}P_{K}}{\sum_{K=I_{a}}^{I_{b}}P_{K}},$$

where: $I_{a}$, $I_{b}$ and $I_{c}$ represent the PSD line index that corresponds to $f_{I_{a}}=0.01\;\mathrm{Hz}$, $f_{I_{b}}=0.2\;\mathrm{Hz}$ and $f_{I_{c}}=2\;\mathrm{Hz}$, respectively.

*Events-related features* characterize the individual and non-superimposed SCRs. They were obtained from the results of the CDA included in two vectors: ${\left\{TON_{K}\right\}}_{K=0\ldots R}$—onset times and ${\left\{AMP_{K}\right\}}_{K=0\ldots R}$—response amplitudes.

**GSR_nimp** represents the number of electrodermal responses detected inside the evaluation window:

(11) GSR_nimp=length(AMP)=length(TON)=R,

**GSR_avimp** represents the average amplitude of electrodermal responses:

(12) $$GSR\_avimp=\frac{1}{R}\overset{R-1}{\underset{K=0}{\sum }}AMP_{k},$$

**GSR_maximp** is the maximum amplitude of the responses inside the evaluation window:

(13) $$GSR\_maximp=\underset{K=0\ldots R-1}{\max}\left(AMP_{K}\right)$$

### 6.4. Heart Rate Variability Features Extraction

The heart rate signal obtained from the plethysmograph optical sensor consisted of a vector $\left\{HR_{K}\right\}$ that contained intervals between successive heart beats.

Seven features were extracted, as follows:

**HR_std** represents the standard deviation of the intervals between successive heart beats inside the evaluation window:

(14) $$HR\_std=\sqrt{\frac{1}{H-1}\overset{H-1}{\underset{K=0}{\sum }}{\left(HR_{K}-\bar{HR}\right)}^{2}},$$

where: H represents the number of heart beats inside the evaluation window and $\bar{HR}$ is the average time between heart beats:

$$\bar{HR}\bar{=}\frac{1}{H}\overset{H-1}{\underset{K=0}{\sum }}HR_{K}$$

**HRV_std** represents the standard deviation of the difference between successive heart rate values inside the evaluation window:

(15) $$HRV\_std=\sqrt{\frac{1}{H-2}\overset{H-1}{\underset{K=1}{\sum }}{\left(HR_{K}-HR_{K-1}-\bar{HRV}\right)}^{2},}$$

where $\bar{HRV}$ is the average time of the heart rate differences:

$$\bar{HRV}=\frac{1}{H-1}\overset{H-1}{\underset{K=1}{\sum }}\left(HR_{K}-HR_{K-1}\right)$$

**HRV_NN50** represents the number of successive heart beat durations that differ by more than 50 ms inside the evaluation window [40]:

(16) $$HRV\_NN50=card\left\{HR_{K}|0.05\leq |HR_{K}-HR_{K-1}|\right\},$$

**HRV_pNN50** is the ratio between the number of successive heart beat durations that differ by more than 50 ms and the total number of heart beats inside the evaluation window [40]:

(17) $$HRV\_pNN50=\frac{HRV\_nn50}{H},$$

**HRV_NN20** represents the number of successive heart beat durations that differ by more than 20 ms inside the evaluation window:

(18) $$HRV\_NN20=card\left\{HR_{K}|0.02\leq |HR_{K}-HR_{K-1}|\right\},$$

**HRV_pNN20** is the ratio between the number of successive heart beat durations that differ by more than 20 ms and the total number of heart beats inside the evaluation window:

(19) $$HRV\_pNN20=\frac{HRV\_nn20}{H}$$

**HRV_hlr** represents the ratio between the *HR* signal energy in the [0.15 Hz,0.4 Hz ] and [0.04 Hz, 0.15 Hz] frequency intervals [41]:

(20) $$HRV\_hIr=\frac{\sum_{K=I_{b}}^{I_{C}}P_{K}}{\sum_{K=I_{a}}^{I_{b}}P_{K}},$$

where: $P_{K}$ represents the -*k*th line of the *HR* signal power spectrum density; $I_{a}$, $I_{b}$ and $I_{C}$ represent the PSD line index that corresponds to $f_{I_{a}}=0.04\;\mathrm{Hz}$; $f_{I_{b}}=0.15\;\mathrm{Hz}$ and $f_{I_{C}}=0.4\;\mathrm{Hz}$, respectively.

## 7. Fear Classification

### 7.1. Machine Learning Methods

#### 7.1.1. Classification Algorithms

There is an increasing interest in the use of artificial intelligence in the field of affective computing. As more as more attention is transferred to the use of automatic emotion recognition, data extraction and data manipulation have become a tipping point in order to achieve good results.

In this section, we unravel a brief analysis of the machine learning and deep learning techniques involved in automatic emotion recognition systems using the biophysical data from the DEAP database.

In order to binarily classify the emotion of fear, we favored the simplest, most popular machine learning methods: SVM, tree-based algorithms—GBT, RF and kNN, as well as shallow and deep artificial neural networks with predefined number of neurons and hidden layers, on which we applied various optimization techniques. Our approach was inspired by Occam’s razor principle [42], which states that the simplest model that fits the data should be preferred. Complex models consume more time and resources and are prone to overfitting [43].

For the non-overlapping dataset, we had a number of 992 samples labeled with 0 (not fear) and 166 samples labeled with 1 (fear). Evidently, the distribution of the observations is very imbalanced. In order to overcome this drawback, the majority class was randomly undersampled, to have 20% more than the number of examples in the minority class, and the minority class was oversampled using the SMOTE technique [44], to have 80% of the number of examples in the majority class. SMOTE is a data augmentation method that synthesizes new examples from the minority class which are close in the feature space, based on the k-Nearest Neighbors (k is usually set to 5). After applying these techniques, we obtained 830 samples labeled with 0 and 664 samples labeled with 1. For the overlapping dataset, initially there were 4960 observations belonging to the 0 class and 830 observations in the 1 class. After randomly undersampling and synthetically oversampling via SMOTE with the same percentages, the proportion turned to 4150/3320.

Cross-validation combines prediction performance from different partitions of the data, which stand for training and validation subsets [43]. This technique reduces variance (the model’s sensitivity to variations in the data), offers an overview of how the model behaves and prevents overfitting. In the case of the k-fold cross-validation, the dataset is split into k equal folds. The model is repetitively trained on k 1-folds and tested on the kth-fold. In the end, after k repetitions, the training and testing scores are averaged.

Feature selection eliminates redundant features, reduces training time, prevents overfitting and can increase classification accuracy, as the model learns from the most relevant features. Dimensionality reduction has the same advantages as feature selection, but it chooses the features from a projected space where relevant and irrelevant features are merged into new ones.

In the case of the SVM and kNN algorithms, the data was scaled, as the features differed by orders of magnitude. These two algorithms require scaling because they involve distances and separation in space. We applied standardization, which removes the mean of the feature and divides by the standard deviation, scaling thus to unit variance. Tree-based algorithms are not sensitive to features at different scales, as they look at each feature independently [43]. As a result, in the case of GBT and RF, scaling was not required.

The following metrics were used:

- The classification report, which presents *precision*, *recall*, *F1 score* and support for both classes. *Precision* computes the proportion of positive identifications which are actually correct. *Recall* (sensitivity or true positive rate) calculates the fraction of true positives that are correctly identified. *F1 score* is the harmonic mean of precision and recall. It is more informative when the dataset is imbalanced [1], as in our case. Support represents the number of occurrences of each class in the dataset, a metric that indicates if the distribution of observations is balanced or imbalanced.

(21) $$precision=\frac{True\;Positives}{True\;Positives+False\;Positives},$$

(22) $$recall=\frac{True\;positives}{True\;positives+False\;Negatives},$$

(23) $$F1\;score=2\cdot \frac{precision\cdot recall}{precision+recall},$$

- The confusion matrix, a table called a contingency table, with two rows and two columns that contain: the rate of observations correctly predicted as negatives (*True Negatives*), the rate of observations incorrectly predicted as positives (*False Positives*), the proportion of observations incorrectly predicted as negatives (*False Negatives*) and the proportion of observations correctly predicted as positives (*True Positives*).
- *Accuracy* calculates the proportion of correct predictions (*True Positives* + *True Negatives*) from the total number of predictions.

(24) $$accuracy=\frac{True\;Positives+True\;Negatives}{True\;Positives+True\;Negatives+False\;Positives+False\;Negatives},$$

- *Specificity* (or true negative rate) measures the proportion of negatives (*True Negatives*) which are correctly identified.

(25) $$specificity=\frac{True\;Negatives}{True\;Negatives+False\;Positives},$$

- The ROC Area Under Curve (ROC AUC) score is a reliable measurement used for binary classification that tells us how much the model distinguishes between classes. Some models can have a high F1 score on average, but a much lower F1 score for one of the classes. A high value of the ROC AUC score means that the model is capable of classifying 0s as 0s and 1s as 1s, so there is an increased rate of True Positives and True Negatives. Acceptable models have an ROC AUC score between 0.7 and 0.8 and those which exceed 0.8 or even 0.9 are considered very solid. The ROC AUC score is optimistic for imbalanced datasets.

The purpose of the SVM algorithm is to find a decision boundary in order to separate data from different classes.

In a space of n dimensions, a hyperplane can be defined by an n-dimensional vector w, and an intercept b. Any data point x on the hyperplane satisfies wx + b = 0. A hyperplane is a separating hyperplane if:

- For any data point x from one class, wx + b > 0.
- For any data point x from another class, wx + b < 0.

The hyperplane is selected so that the distance between the nearest points in each class (called support vectors) and the hyperplane is maximal. When it is impossible to segregate a set of observations containing outliers, their misclassification is permitted, but the error they introduce should be minimized. The penalty hyperparameter C controls misclassification and the strictness of separation. A large C penalizes misclassification, leading to overfitting. If C is small, more data points will be misclassified, introducing a high bias. The kernels solve non-linear classification problems by transforming the original feature space into a linearly separable dataset. The Radial Basis Function (RBF) or Gaussian kernel presents the hyperparameter γ, the kernel coefficient, which determines how strictly the kernel function fits the data. A large γ produces overfitting, while a small γ leads to underfitting. The scikit-learn library [45] uses as default for γ the value 1/number_of_features. SVM can achieve a high accuracy of classification with the appropriate kernel and parameters [43].

We used the C-Support Vector Classification (SVC) function from the scikit-learn library. Considering the number of instances and features (the number of instances is not significantly larger than the number of features), the RBF kernel was the most appropriate choice. Our dataset is imbalanced, so we set class weight = “balanced” to emphasize the underrepresented classes.

The learning curve is a graph that compares the cross-validated training and testing scores. It is used to detect overfitting or underfitting. When the testing score converges at a much lower accuracy than the training score, below the desired performance, we conclude that there is a situation of overfitting. Thus, the model fits too well on the training set and fails to generalize on the testing set. When both the training and the testing scores are well below the desired performance, underfitting occurs. As such, the model is unreliable and incapable of capturing the underlying relationship in the data. We performed a five-fold cross-validation procedure with a testing (validation) batch of 30% of the data and plotted the training and testing scores against the number of training examples, as well as the scalability of the model—fit times (times spent for fitting in seconds) against the number of training examples and performance of the model—testing score versus fit times.

For a number of 10 iterations (10 rounds), we split the original dataset into training and testing subsets (70% training and 30% testing), using the train_test_split function from the scikit-learn library. This function randomly splits the data, preserving the percentage of observations for each class. The functionality of GridSearchCV from scikit-learn implies data splitting, fold generation, cross training and validation and exhaustive search over the best set of parameters, maximizing classification accuracy. We applied a five-fold cross-validation on the training subset that ran in parallel on all available cores. The values we tested for the penalty C were: {0.1,1,10,100,1000,10000} and for the kernel coefficient γ: {1/number_of_features, 1/number_of_features ∗ 10, 1/number_of_features ∗ 100, 1/number_of_features ∗ 1000}. The variable number_of_features represents the number of features (120 features for the non-overlapping dataset and 40 features for the overlapping one). The most optimized estimator (the model with the best set of hyperparameters that maximized classification accuracy on the training subset) was validated on the testing subset. The resulting F1 score, ROC AUC score, accuracy, sensitivity and specificity were calculated at each iteration and on average, for all the 10 iterations. Confusion matrix and classification report were also generated at each iteration.

#### 7.1.2. Dimensionality Reduction and Feature Selection Algorithms

PCA is a dimensionality reduction technique which increases the classifier’s performance. We projected the original data into a 100-dimensional space for the non-overlapping dataset and into a 20-dimensional space for the overlapping dataset, followed by cross-validation, the plotting and assessment of the learning curves, splitting into training and testing subsets, grid search for the best parameters on the training subset and classification using the SVC classifier with the RBF kernel on the testing subset throughout 10 iterations, as previously described. The same metrics were computed for each of the 10 iterations and on average for all 10.

In order to perform feature selection, we applied various algorithms: XGBoost, Pearson Correlation Coefficient, L1 regularization, RF Classification and Recursive Feature Elimination.

For each feature selection algorithm, we repetitively chose the first k most relevant features. The variable k takes the values: 5, 10, 20, 30, 40, 50, 60, 70, 80, 90, 100, 110 and 120 for the non-overlapping dataset and 5, 10, 20, 30 and 40 for the overlapping dataset. For each feature selection algorithm, for each k, we undertook the following steps:

- We split the original dataset into training and testing subsets (70% training and 30% testing), using the train_test_split function from the scikit-learn library.
- We applied a five-fold cross-validation on the training subset that ran in parallel on all available cores. The values we tested for the penalty C were: {0.1, 1, 10, 100, 1000, 10,000} and for the kernel coefficient γ: {1/number_of_features, 1/number_of_features ∗ 10, 1/number_of_features ∗ 100, 1/number_of_features ∗ 1000}. The variable number_of_features represents the number of features, which is equal to k.
- The most optimized estimator (the model with the best set of hyperparameters that maximized classification accuracy on the training subset) was validated on the testing subset, computing the resulting confusion matrix, classification report, F1 score, ROC AUC score, accuracy, sensitivity and specificity.

RF is an ensemble of trees (called tree bagging) that outperforms simple DT by reducing variance and preventing overfitting. The individual trees that are included in the forest are trained on different sets of features. For improving the quality of feature and value separation at the splitting point we have used the Gini criterion and then tuned the other hyperparameters [43]:

- max_depth—the maximum depth of an individual tree. We picked three options: {3, 10, None}. None means that the nodes are expanded until all leaves are pure or until all leaves contain less than min_samples_split samples.
- min_samples_split—the minimum number of samples required for node splitting. A small value can cause overfitting and a large one leads to underfitting. The values to explore for this hyperparameter were: {10, 30, 50, 70, 90}.
- max_features—the number of features required for best node splitting in an individual tree. The options we considered were sqrt(number_of_features) and log2(number_of_features).
- n_estimators—the number of trees required for majority voting in the bagging algorithm after the individual trees have been trained separately. More trees lead to better classification rates. The options we tweaked were {100, 300, 500}.

We performed the five-fold cross-validation procedure and plotted the learning curves, model scalability and model performance. Similarly to the approach described for SVM, the data were split into training and testing subsets for 10 rounds, we grid searched for the most optimal parameters on the training subset and computed the metric scores after validating the best resulting model on the testing subset.

In the case of GBT, the trees are built and trained in succession, in an iterative way, each new tree trying to correct the residual errors introduced in the prediction by the previous trees. We set the learning rate (or shrinkage factor, which controls the weighting of new trees added to the model) at 0.1, the max_depth = 10 and n_estimators = 1000. We performed the five-fold cross-validation procedure and plotted the learning curves, model scalability and model performance. The data were split into training and testing subsets for 10 times (70% training and 30% testing) and we computed the metric scores after validating the best resulting model on the testing subset. In the case of GBT, we did not have to tune the parameters using grid search because we chose a fixed set of parameters from the start.

For both RF and GBT, there was no need to scale the data using the standard scaler nor to perform feature selection.

The kNN algorithm predicts the output by finding the nearest neighbor class. It is calculated as the most common class among the k-Nearest Neighbors.

We performed a five-fold cross-validation procedure with a testing (validation) batch of 30% of the data and plotted the training and testing scores, model scalability and performance. For cross-validation, the number of nearest neighbors was set to five. For 10 times, we split the original dataset into training and testing subsets (70% training and 30% testing). The parameters of the KNeighborsClassifier function from the scikit-learn library we grid searched for were: leaf_size (in the range 5 to 50), n_neighbors (in the range 3 to 9) and p (in the range 1 to 2). p is the power parameter for the Minkowski metric. *p* = 1 represents the Manhattan distance and *p* = 2 stands for the Euclidean distance. The most optimized estimator (the model with the best set of parameters that maximized classification accuracy on the training subset) was validated on the testing subset. The resulting F1 score, ROC AUC score, accuracy, sensitivity and specificity were calculated at each iteration and, on average, for all 10 iterations. Confusion matrix and classification report were also generated at each iteration. Similarly to the approach described for SVM, we performed dimensionality reduction using PCA and feature selection by applying the XGBoost, Pearson Correlation Coefficient, L1 regularization and RF Classification algorithms. Unfortunately, the Recursive Feature Elimination technique was too computationally expensive and failed to run effectively, so we dropped it from the final evaluation.

### 7.2. Deep Learning Methods

The experimentation process was represented by a series of trials of several network configurations. We tested on different architectures for the neural networks, either shallow by employing only the input and the output layers, or deep by adding other hidden layers. The tuning of hyperparameters (the number of hidden layers, the number of neurons, the learning rate, the number of epochs and batch size) required the utmost attention in order to evaluate how these could affect or improve the quality of our neural networks and how resilient the data are against changes in the network’s configuration.

The framework we used for implementing the networks was Keras [46] in the Tensorflow environment [47]. Furthermore, we used the scikit-learn library to process the data and to compute the classification scores. The matplotlib library [48] was employed to generate trustworthy plots that were able to generate a better and more comprehensive understanding of the model’s performance.

As the rule of thumb states that the input data has to be scaled, we used the scikit-learn StandardScaler function to fit and transform it.

We closely followed two approaches:

- We used the train_test_split function from the scikit-library with a fixed seed for the random_state variable with the aim to always obtain the same, predictable slices from the input and the label arrays for the training and testing sessions, averaging the results for 10 rounds.
- We performed a five-fold cross-validation which is beneficial in the case of overfitting.

We used and customized a Python program for fine tuning our neural network as presented in [43] and displayed the results in Tensorboard [49] in order to reveal the best mix of hyperparameters that influenced the dynamic of our program, such as the number of neurons on the hidden layers, the right values for dropouts for different layers, the number of epochs and the best values for the learning rate. A rule of thumb method states that the number of neurons on the hidden layer should be 2/3 the size of the input layer, plus the size of the output layer [50]. Another one mentions that the number of neurons on the hidden layer should be the equal to the square root of the product between the size of the input layer and that of the output layer. For the non-overlapping dataset, we configured: the number of neurons on the hidden layer (80 representing two thirds of the number of neurons on the input layer and 11 being the square root of the product between the number of neurons on the input and output layers), the number of epochs over which the network has to train (100 or 200), the dropout rate (0.3 or 0.4 or 0.5) for the input layer and the learning rate (with values between 0.01 and 0.2). The neural network was a simple one, with 120 neurons on the input layer (equal to the number of input features), a hidden layer with the ReLU activation function and an output layer with one neuron. For the overlapping dataset, we configured: the number of neurons on the hidden layer (27 and 6), the right values for dropouts for different layers (0.3, 0.35, 0.4, 0.5), the number of epochs (100 or 200) and the best value for the learning rate (between 0.01 and 0.2). The network had 40 neurons on the input layer, equal to the number of input features.

## 8. Results

### 8.1. Results of the Machine Learning Algorithms

#### 8.1.1. Results for the Non-Overlapping Dataset

Table 1 presents the results of the SVM algorithm, applied on the non-overlapping dataset. The highlighted values represent the highest scores obtained.

**Figure 6:**
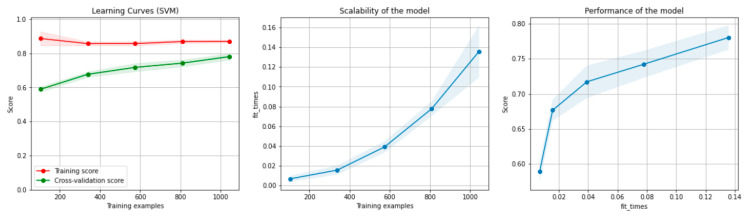
Learning curves, model scalability and model performance for the SVM algorithm, for the non-overlapping dataset.

**Figure 7:**
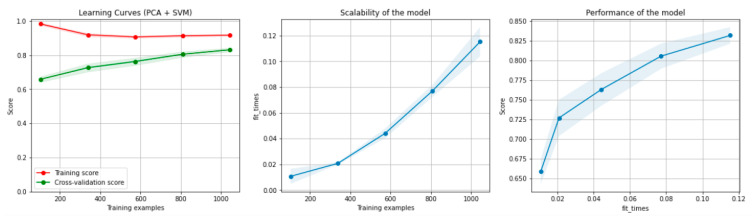
Learning curves, scalability and model performance for the PCA + SVM algorithm, for the non-overlapping dataset.

Figure 6 and Figure 7 present the learning curves, model scalability and model performance for the SVM and PCA dimensionality reduction + SVM algorithms applied on the non-overlapping dataset. The cross-validation score tends to converge to the training score, above a desired performance of 75 (SVM) and 80% (PCA dimensionality reduction + SVM), respectively, which implies that there is no situation of overfitting. Additionally, the model scalability and model performance have an ascending pattern over an increasing number of training examples and fit times.

Table 2 presents the results of the DT algorithms, applied on the non-overlapping dataset. The highlighted values represent the highest scores obtained.

**Figure 8:**
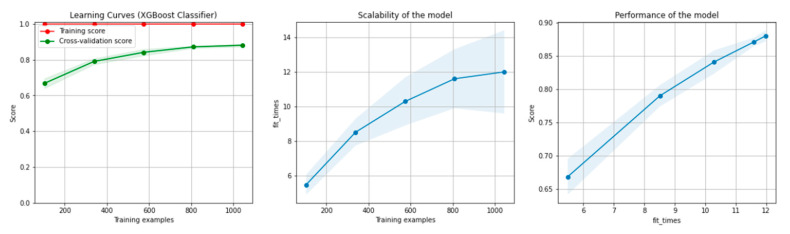
Learning curves, model scalability and model performance for the Gradient Boosting Tree algorithm, for the non-overlapping dataset.

**Figure 9:**
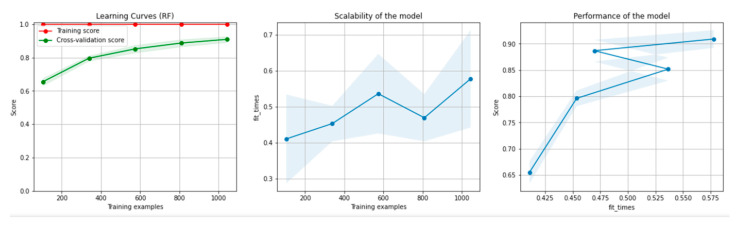
Learning curves, scalability and model performance for the RF algorithm, for the non-overlapping dataset.

Figure 8 and Figure 9 present the learning curves, model scalability and model performance for the Gradient Boosting Trees and RF algorithms applied on the non-overlapping dataset. The cross-validation score tends to converge to the training score, above a desired performance of 80%, which implies that there is no situation of overfitting. Additionally, the model scalability and model performance have an ascending pattern over an increasing number of training examples and fit times.

Table 3 presents the results of the kNN algorithm, applied on the non-overlapping dataset. The highlighted values represent the highest scores obtained.

**Figure 10:**
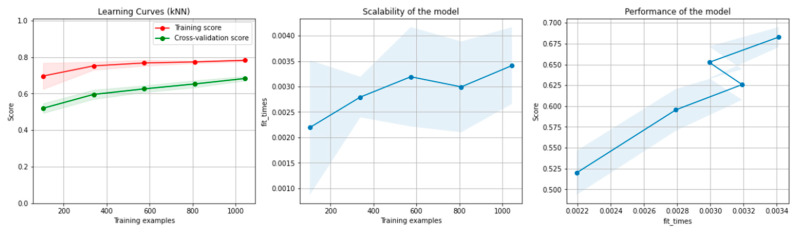
Learning curves, scalability and model performance for the kNN algorithm, for the non-overlapping dataset.

**Figure 11:**
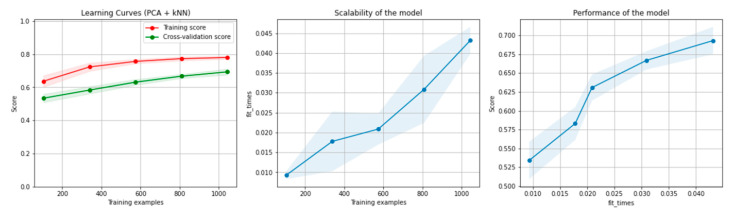
Learning curves, scalability and model performance for the PCA + kNN algorithm, for the non-overlapping dataset.

Figure 10 and Figure 11 present the learning curves, model scalability and model performance for the kNN and PCA dimensionality reduction + kNN algorithms applied on the non-overlapping dataset. The cross-validation score tends to converge to the training score, above a desired performance of 70%, which implies that there is no situation of overfitting. Additionally, the model scalability and model performance have an ascending pattern over an increasing number of training examples and fit times.

Table 4 presents the top 10 features, according to their importance, which were selected by the SVM and the kNN algorithms for the non-overlapping dataset. [W1] corresponds to the first window of the trial (the first 20 s), [W2]—the second window of the trial (seconds 21–40) and [W3]—the third window (seconds 41–60).

#### 8.1.2. Results for the Overlapping Dataset

Table 5 presents the results of the SVM algorithm, applied on the overlapping dataset. The highlighted values represent the highest scores obtained.

**Figure 12:**
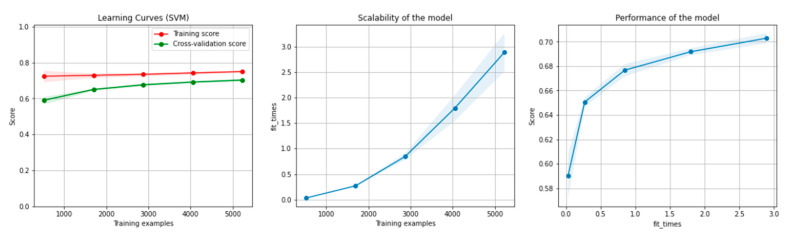
Learning curves, model scalability and model performance for the SVM algorithm, for the overlapping dataset.

**Figure 13:**
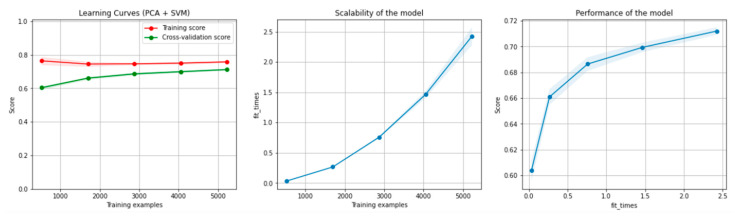
Learning curves, scalability and model performance for the PCA + SVM algorithm, for the overlapping dataset.

Figure 12 and Figure 13 present the learning curves, model scalability and model performance for the SVM and PCA dimensionality reduction + SVM algorithms applied on the overlapping dataset. The cross-validation score tends to converge to the training score, above a desired performance of 70%, which implies that there is no situation of overfitting. Additionally, the model scalability and model performance have an ascending pattern over an increasing number of training examples and fit times.

Table 6 presents the results of the DT algorithms, applied on the overlapping dataset. The highlighted values represent the highest scores obtained.

**Figure 14:**
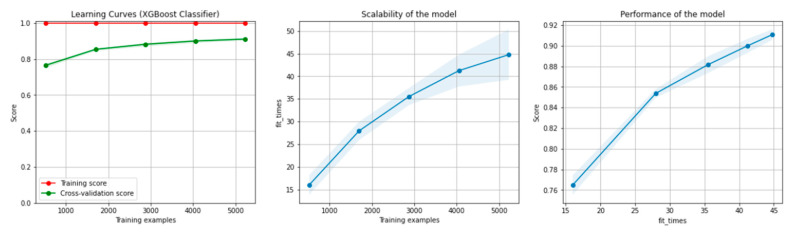
Learning curves, model scalability and model performance for the GBT algorithm, for the overlapping dataset.

**Figure 15:**
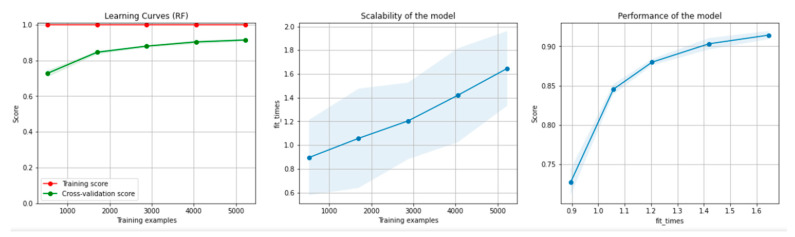
Learning curves, scalability and model performance for the RF algorithm, for the overlapping dataset.

Figure 14 and Figure 15 present the learning curves, model scalability and model performance for the GBT and RF algorithms applied on the overlapping dataset. The cross-validation score tends to converge to the training score, above a desired performance of 80%, which implies that there is no situation of overfitting. Additionally, the model scalability and model performance have an ascending pattern over an increasing number of training examples and fit times.

Table 7 presents the results of the kNN algorithm, applied on the overlapping dataset. The highlighted values represent the highest scores obtained.

**Figure 16:**
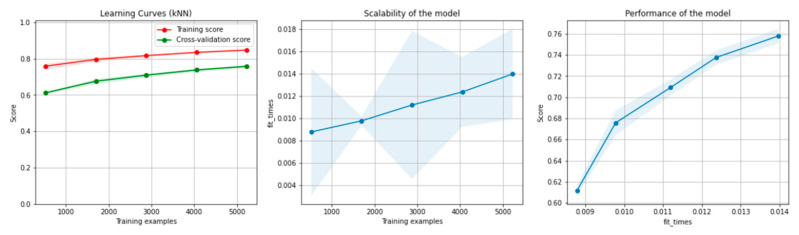
Learning curves, scalability and model performance for the kNN algorithm, for the overlapping dataset.

**Figure 17:**
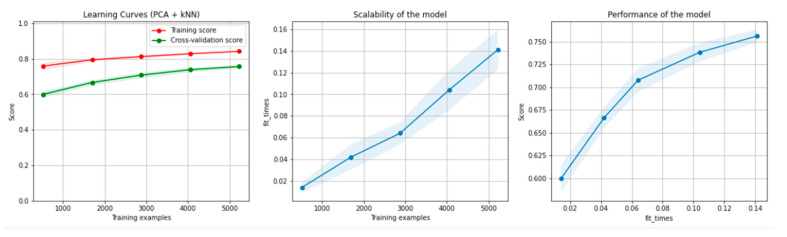
Learning curves, scalability and model performance for the PCA + kNN algorithm, for the overlapping dataset.

Figure 16 and Figure 17 present the learning curves, model scalability and model performance for the kNN and PCA dimensionality reduction + kNN algorithms applied on the overlapping dataset. The cross-validation score tends to converge to the training score, above a desired performance of 75%, which implies that there is no situation of overfitting. Additionally, the model scalability and model performance have an ascending pattern over an increasing number of training examples and fit times.

Table 8 presents the top 10 features, according to their importance, which were selected by the SVM and the kNN algorithms for the overlapping dataset.

### 8.2. Results for the Artificial Networks Configurations

#### 8.2.1. Results for the Non-Overlapping Dataset

Tensorboard selected as the best configuration to maximize classification accuracy (82.5%) the one with 80 neurons on the hidden layer, a dropout of 0.3 and learning rate of 0.01, run over 200 epochs. In order to prevent overfitting, we designed five configurations starting from this one and plotted the learning curves (Table 9).

We tested the following configurations with 120 neurons on the input layer, 1 neuron on the output layer, the ReLU activation function and a batch size of 32.

**Figure 18:**
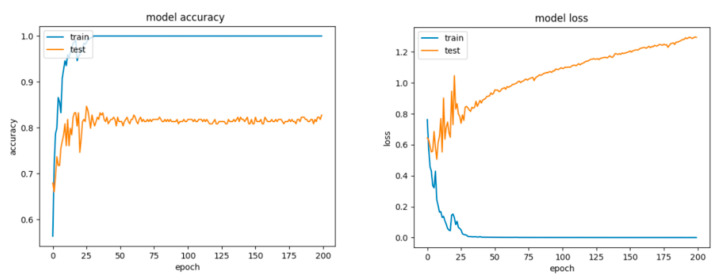
Accuracy and loss for Configuration 1, non-overlapping dataset.

For Configuration 1, as we expected, omitting any regularization implied the occurrence of overfitting during cross-validation with 70% of training and 30% of testing data. Moreover, one of the first things that stands out while looking at the learning curves presented in Figure 18 is how drastically the basic neural network’s dynamic changed over the epochs.

A good fit means that the training and validation loss decrease to a point of stability (lower loss on the training dataset than on the validation dataset) with a minimal gap (called “generalization gap”) between the two final loss values.

**Figure 19:**
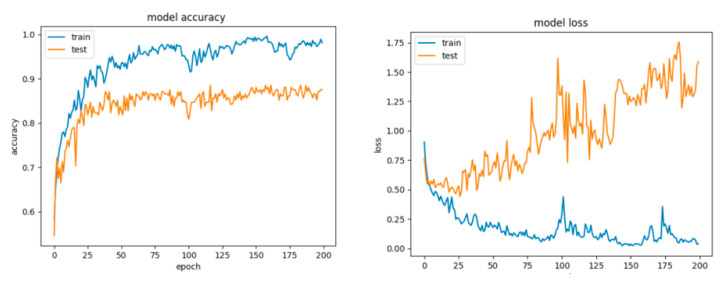
Accuracy and loss for Configuration 2, non-overlapping dataset.

For Configuration 2, introducing a dropout strategy reduced partially, but not sufficiently, the overfitting effect (Figure 19) and yielded comparably good results as well (Table 10). Furthermore, by running multiple tests with an even greater dropout, of 0.8, we obtained similar scores.

**Figure 20:**
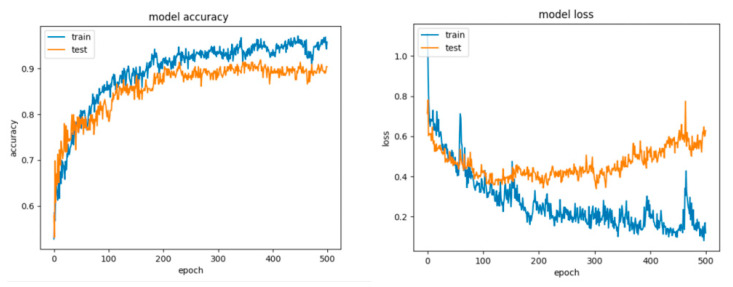
Accuracy and loss for Configuration 3, non-overlapping dataset.

The learning curves for Configuration 3 are presented in Figure 20.

**Figure 21:**
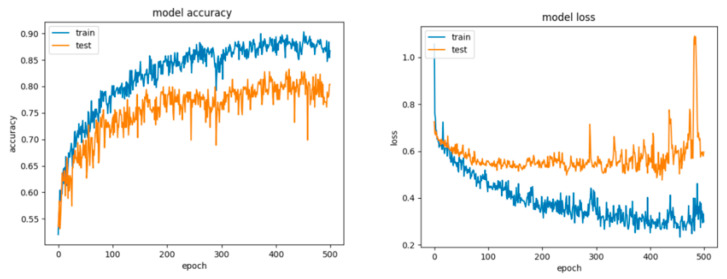
Accuracy and loss for Configuration 4, non-overlapping dataset.

For Configuration 4, it can be observed that the extra layer did not improve performance; however, the scores are similar to those obtained for the previous configurations (Table 10). Despite this fact, looking at the curves for loss and accuracy, it can be inferred that the model had a tendency to overfit (Figure 21).

**Figure 22:**
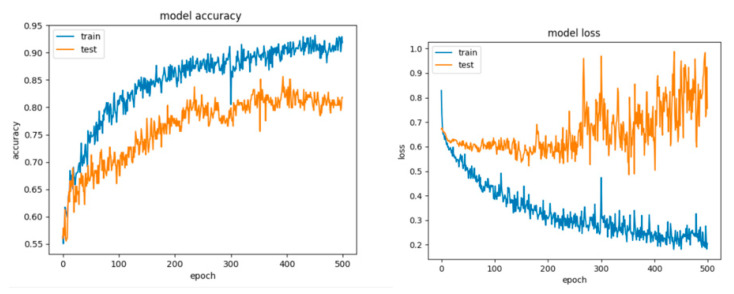
Accuracy and loss for Configuration 5, non-overlapping dataset.

For Configuration 5, we considered changing the number of neurons on the hidden layer to 11, to match the formula presented in [51]. It can be observed that adding the extra layer did not help improve the performance (Table 10); however, the model still tended to overfit (Figure 22).

We chose Configuration 3 to be the best model because it did not overfit and because it also obtained some of the best classification scores (ROC AUC score of 86.1%). The five-fold cross-validation (during 500 epochs with a batch size of 32, Adam Optimizer with a decaying learning rate) and the averaged scores for 10 runs for this model (70% training and 30% testing data) are presented in Table 11.

#### 8.2.2. Results for the Overlapping Dataset

Tensorboard selected as the best configuration to maximize classification accuracy (79.2%) the one with six neurons on the hidden layer, a dropout of 0.3 and learning rate of 0.01, run over 200 epochs. In order to prevent overfitting, we designed seven configurations starting from this one and plotted the learning curves (Table 12).

We tested the following configurations with 40 neurons on the input layer, 1 neuron on the output layer, the ReLU activation function and a batch size of 32.

**Figure 23:**
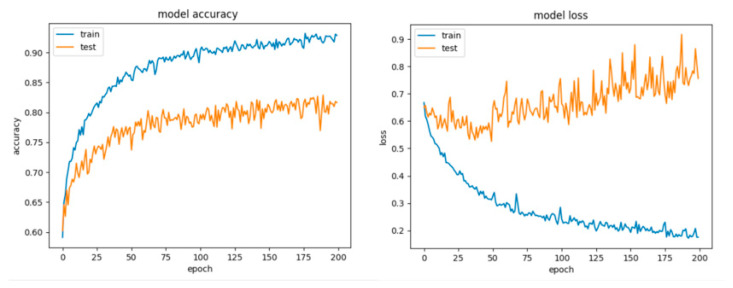
Accuracy and loss for Configuration 1, overlapping dataset.

By looking at the learning curves for Configuration 1 (Figure 23), the considerable discrepancy between the accuracy and loss scores obtained during the training session with respect to their counterparts obtained during the testing session (the data has been split into 70% training and 30% testing) can be easily observed. Despite the fact that with this configuration we achieved good scores for the training session (accuracy over 90% and little loss), it can be inferred that the network learned too well and had the tendency to overfit the data, thus resulting in a poor performance on the testing set.

Henceforth, we established using dropouts as a means of regularization to reduce overfitting.

**Figure 24:**
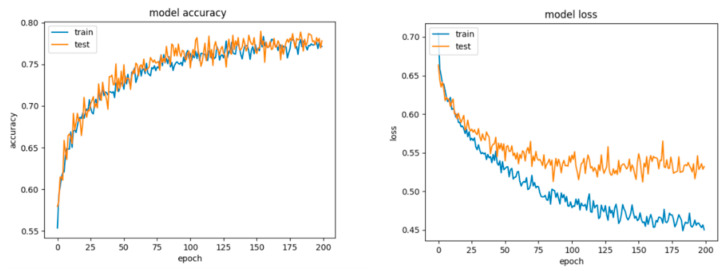
Accuracy and loss for Configuration 2, overlapping dataset.

As it can be observed for Configuration 2 (Figure 24, Table 13), the network performed slightly worse, misclassifying more examples of both classes, this being a consequence of including the regularization procedure. Nevertheless, the gap between the accuracy and loss for the train and test diminished and the model did not overfit anymore. The first question that arises is the legitimate choice of the dropout value. Consequently, we settled for a dropout of 0.5.

For Configuration 3, we considered letting the neural network train slightly longer to envision whether it would hit a performance plateau or yield better results. Thus, we trained for 500 epochs with no dropout.

Notwithstanding the fact that training accuracy reached beyond the value of 95% as compared to the accuracy obtained for Configuration 1 with only 200 epochs, the test accuracy limited itself to almost the same value (Table 13). On the other hand, the loss in this case skyrocketed and diverged even more.

For Configuration 4, we introduced dropout in order to reduce overfitting and trained for 500 epochs.

The testing results came closer to the training results; however, both of them were lower due to dropping random nodes from the net. The conclusion we drew was to settle for a number of epochs of 200 and to further change the architecture, as more training did not effectively help the network to generalize better.

For Configuration 5, we added a hidden layer with six neurons, which did not improve the overall performance.

We chose the next configuration (Configuration 6) to have one hidden layer with 27 neurons and another one with six neurons, according to [50,51]. In this situation, the testing scores were even lower (Table 13).

For Configuration 7, we designed a network with three hidden layers with 30, 20 and 10 neurons on each of them.

We chose Configuration 2 to be the best model because it did not overfit and because it also obtained some of the best classification scores (ROC AUC score of 77.4%). The five-fold cross-validation (over 200 epochs with a batch size of 32, Adam Optimizer with a decaying learning rate) and the averaged scores for 10 runs (70% training and 30% testing data) for this model are presented in Table 14.

## 9. Discussion

### 9.1. Discussion of the Results for the Non-Overlapping Dataset

In order to see whether the algorithms (simple algorithm, with dimensionality reduction and with feature selection) applied on the data had the same efficiency, we performed the one-way ANOVA test for independent measures. With regard to the results of the SVM algorithm (Table 1), we obtained that there was no significant difference between the sets of cross-validation grid search test score, F1 score, ROC AUC score, accuracy, sensitivity and specificity (*p* = 0.48, F = 0.92), which means that all the methods had the same efficacy (the averages of all groups were equal) for SVM, PCA + SVM, XGBoost Feature Selection + SVM, Pearson Feature Selection + SVM, L1 Regularization Feature Selection + SVM, Random Forest Classification Feature Selection + SVM and Recursive Feature Elimination Feature Selection + SVM. Additionally, there was no difference between the averages of the F1 score, ROC AUC score, accuracy, sensitivity and specificity over 10 iterations for the methods SVM and PCA dimensionality reduction + SVM (*p* = 0.51, F = 0.47). Table 15 presents the *p* and F values for the SVM, DT and kNN algorithms. The results show that there is no statistical difference between the classification scores obtained with and without feature selection and dimensionality reduction. Practically all tested methods resulted in the same classification performance.

The highest ROC AUC score was obtained for the PCA dimensionality reduction + SVM algorithm (93.5%, C = 1000, gamma = 0.083 (1/n_features ∗ 10)). The simple SVM algorithm led to an ROC AUC score of 92.7% (C = 10, gamma = 0.083, (1/n_features ∗ 10)). In order to select fewer features, we applied the Recursive Feature Elimination Feature Selection + SVM method with the top 40 features (C = 1000, gamma = 0.25 (1/n_features ∗ 10)) and obtained an ROC AUC score of 92.7%.

The GBT and RF algorithms resulted in similar ROC AUC scores (90 and 89.4%, respectively).

With regard to the kNN algorithm, the highest ROC AUC score was achieved for the XGBoost Feature Selection + kNN (81.4%), with the top 30 features, leaf_size = 5, n_neighbors = 3 and *p* = 1. With PCA dimensionality reduction, we obtained an ROC AUC score of 79.6% (leaf_size = 42, n_neighbors = 4, *p* = 1) and by applying only kNN, an ROC AUC score of 80.85% (leaf_size = 5, n_neighbors = 4, *p* = 1).

We chose Configuration 3 to be the best model, because it did not overfit and because it also obtained some of the best classification scores (ROC AUC score of 86.1%).

To sum up, the highest ROC AUC scores were obtained, in order, for PCA dimensionality reduction + SVM—93.5%, GBT—90%, an artificial neural network with no hidden layers and 0.8 dropout on the input layer—86.1% and the XGBoost Feature Selection + kNN, with the top 30 features—81.4%. If we want to select fewer features and to have at the same time a high classification performance, Recursive Feature Elimination Feature Selection + SVM algorithm with the top 40 features led to an ROC AUC score of 92.7%.

### 9.2. Discussion of the Results for the Overlapping Dataset

Table 16 presents the *p* and F values for the SVM, DT and kNN algorithms. The results show that there is no statistical difference between the classification scores obtained with and without feature selection and dimensionality reduction for SVM, DT and kNN grid search. Only by testing the best resulting model over 10 iterations with the kNN algorithm did we obtain better classification scores without PCA dimensionality reduction than by using PCA dimensionality reduction (*p* = 0.006).

The highest ROC AUC score was obtained for the XGBoost Feature Selection + SVM with all 40 features (89.2%, C = 10, gamma = 0.25 (1/n_features ∗ 10)). The simple SVM algorithm led to an ROC AUC score of 88.8% (C = 10, gamma = 0.25, (1/n_features ∗ 10)) and with PCA dimensionality reduction an ROC AUC score of 86% (C = 10, gamma = 0.25, (1/n_features ∗ 10)).

The GBT and RF algorithms resulted in similar ROC AUC scores (91.7 and 89.6%, respectively).

In what concerns the kNN algorithm, the highest ROC AUC score was achieved by applying the kNN algorithm only, without dimensionality reduction and without feature selection—83.9%, with leaf_size = 5, n_neighbors = 4 and *p* = 1. With PCA dimensionality reduction, we obtained an ROC AUC score of 78.7% (leaf_size = 6, n_neighbors = 4, *p* = 1) and by applying feature selection, with the top 30 features, either through XGBoost Feature Selection or Random Forest Classification Feature Selection, we achieved an ROC AUC score of 82.5% (leaf_size = 5, n_neighbors = 3, *p* = 1). Over 10 iterations, the best kNN model (leaf_size = 5, n_neighbors = 4, *p* = 1) obtained a higher ROC AUC score (81.8%) without PCA dimensionality reduction that was statistically significant at *p* = 0.001.

We chose Configuration 2 to be the best model because it did not overfit and because it also obtained some of the best classification scores (ROC AUC score of 77.4%).

In conclusion, the highest ROC AUC scores were obtained, in order, for GBT—91.7%, SVM—89.2%, kNN—83.9% and an artificial neural network with no hidden layers and 0.5 dropout on the input layer—77.4%. If we want to select fewer features and to have at the same time a high classification performance, XGBoost Feature Selection or Random Forest Classification Feature Selection + kNN with the top 30 features led to an ROC AUC score of 82.5%.

Concerning the SVM algorithm, the best performing model on the non-overlapping dataset (which was PCA dimensionality reduction + PCA) was more efficient than the best model on the overlapping dataset (which was XGBoost feature selection + SVM, where all 40 features were selected)—*p* = 0.0004, F = 26.7. The same pattern was valid for the artificial network configurations—Configuration 3 for the non-overlapping dataset performed better than Configuration 2 for the overlapping dataset (*p* = 0.0002, F = 40.92).

For the DT, the best-performing algorithms were GBT for both non-overlapping and overlapping datasets. Their efficiency was, however, similar (*p* = 0.47, F = 0.57).

For the kNN algorithm, the best-performing model on the non-overlapping dataset (XGBoost feature selection + kNN, with the top 30 features) was more efficient than the best model on the overlapping dataset (kNN only)—*p* = 0.32, F = 1.06.

### 9.3. Discussion of Feature Selection

By applying the SVM and kNN classification algorithms using the two approaches (non-overlapping and overlapping), we arrived at four sets of features considered as the most relevant in terms of their importance in the classification process.

In the case of the non-overlapping approach, by analyzing the top 10 features obtained by means of the two algorithms, we observed that six of them were common: HRV_hlr, SCR_fmd, SCL_fmd, GSR_fmd, HRV_NN50 and HRV_NN20. This observation confirms the consistency of the selection process and indicates that these features have the highest degree of correlation with fear.

In the case of the overlapping approach, the number of common features selected for the two algorithms was higher, with nine in the top 10—HRV_hlr, SCR_fmd, SCL_fmd, GSR_fmd, HRV_NN50, HRV_NN20, GSR_mav, SCL_mav and GSR_nimp—which means that this approach led to more relevant features for the classification process.

It was also observed that all features selected using the non-overlapping approach were found in the set of features selected using the overlapping approach. This indicates that these features are very relevant, being the ones that carry most of the information needed to classify fear. In reality, there are three categories of basic features: median frequencies of the power spectrum (fmd), the ratio between the signal energy in the high- and low-frequency intervals (hlr) and the number of successive heart beat durations (NN).

fmd is a relevant parameter for SCR and SCL, but also for GSR. For SCR, a higher value of the median frequencies of the power spectrum could be explained by the presence of a higher number of NS-SCR. As the increase in NS-SCR frequency is correlated with fear [22], fmd is a relevant parameter from this point of view. An increasing SCL has higher median frequencies of the power spectrum than a constant SCL, fmd thus providing information on the baseline anxiety state. There is therefore a correlation between the fmd value and fear, an increase in the frequency of this parameter indicating a variable stress level. A lower value of SCL_fmd corresponds to a more constant SCL level, being an indicator of relaxation. GSR_fmd is correlated with the distribution of high and low frequencies depending on the energy level.

NN, the number of high differences between successive NN intervals, is correlated with high frequency power, reflecting the vagal (PNS) activity [17,52], an indicator of the ability to adapt to stress.

hlr, the ratio of high-frequency to low-frequency power, reflects the balance between the sympathetic and vagal activity. It has been applied in an attempt to better estimate the sympathetic activity [17,52].

The most relevant features are part of the categories fmd, hlr and NN, which are related to the GSR signal’s phasic and tonic components, as well as to the HRV intervals. It is intuitive that they are important and that they contain fear-related information, thus making the classification algorithms more predictable and trustworthy in the decision-making process.

### 9.4. Predictions Interpretation Using the LIME Method

In order to understand why the models behave in a certain way, we applied the LIME method for interpretation and for explaining predictions in a human-understandable manner [53].

LIME is model-agnostic, as it is able to explain any model without making assumptions about it, provides a human-understandable and interpretable representation so that the user is provided with a global intuition of the model and is locally faithful for any particular data point that replicates the model’s behavior [54].

**Figure 25:**
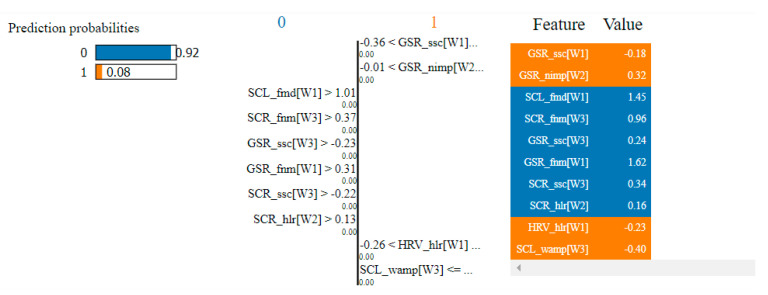
Prediction features for situation 0 (no fear), for the non-overlapping dataset.

After applying the SVM algorithm and the LIME method on the non-overlapping dataset, the prediction interpretation for a particular data sample is presented in Figure 25. The model was 92% confident that the prediction was 0. The features colored in blue (SCL_fmd[W1], SCR_fnm[W3], GSR_ssc[W3], GSR_fnm[W1], SCR_ssc[W3], SCR_hlr[W2]) increase the chances to predict the output value of 0, while those colored in orange decrease it (increase the chances to predict the output value of 1).

**Figure 26:**
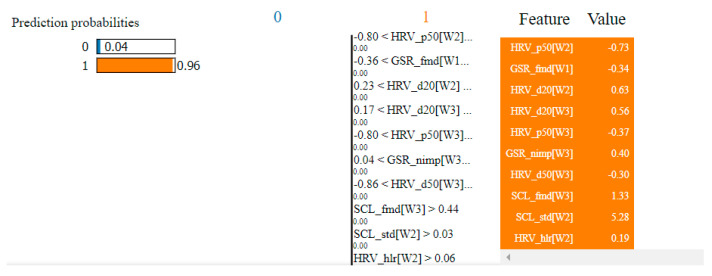
Prediction features for situation 1 (fear), for the non-overlapping dataset.

In Figure 26, the model is 96% confident that the prediction is 1. The features colored in orange (HRV_p50[W2], GSR_fmd[W1], HRV_d20[W2], HRV_d20[W3], HRV_p50[W3], GSR_nimp[W3], HRV_d50[W3], SCL_fmd[W3], SCL_std[W2], HRV_hlr[W2]) increase the chances to predict the output value of 1.

The features SCL_fmd[W1], GSR_fmd[W1] and SCL_fmd[W3] were selected in the top 10 most relevant features by the feature selection algorithms (Table 4).

**Figure 27:**
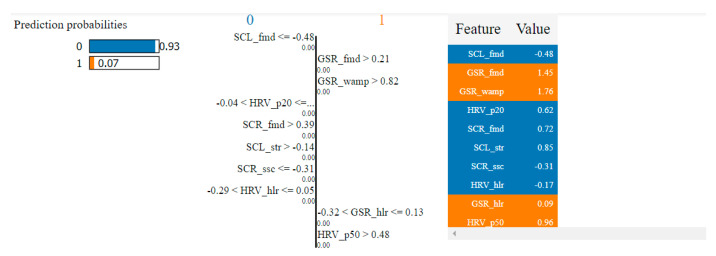
Prediction features for situation 0 (no fear), for the overlapping dataset.

After applying the SVM algorithm and the LIME method on the overlapping dataset, the prediction interpretation for a particular data sample is presented in Figure 27. The model was 93% confident that the prediction was 0. The features colored in blue (SCL_fmd, HRV_p20, SCR_fmd, SCL_str, SCR_ssc, HRV_hlr) increase the chances to predict the output value of 0, while those colored in orange decrease it (increase the chances to predict the output value of 1).

**Figure 28:**
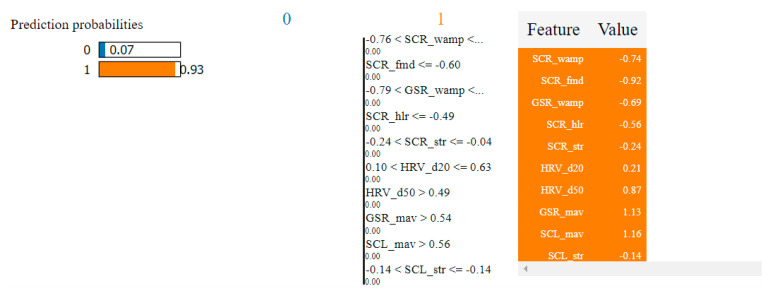
Prediction features for situation 1 (fear), for the overlapping dataset.

In Figure 28, the model is 93% confident that the prediction is 1. The features colored in orange (SCR_wamp, SCR_fmd, GSR_wamp, SCR_hlr, SCR_str, HRV_d20, HRV_d50, GSR_mav, SCL_mav, SCL_str) increase the chances to predict the output value of 1.

The features SCL_fmd, SCR_fmd, HRV_hlr, GSR_mav and SCL_mav were selected in the top 10 most relevant features by the feature selection algorithms (Table 8).

### 9.5. Comparison with Similar Studies

Our results are better than those obtained by Machajdik et al. [24], who used heart rate data to classify fear. They obtained a classification accuracy of 91.47% using DT. For the overlapping dataset, our GBT algorithm reached an ROC AUC score of 91.7%, while for the non-overlapping dataset, 93.5% using SVM and 92.7% using Recursive Feature Elimination for Feature Selection and SVM.

Pan et al. [28] classified the emotional state characterized by low valence and high arousal based on the physiological features from the DEAP dataset with an average classification accuracy of 76.46% using a fusion of SVM and DT. Our SVM and DT algorithms outperformed this result with more than 12% for the ROC AUC score for both the overlapping and non-overlapping dataset.

Our results are better than those obtained by Dominguez et al. [1], who classified sadness based in PPG and GSR with an accuracy of 91% using Recursive Feature Elimination for Feature Selection and SVM. In our case, Recursive Feature Elimination and SVM resulted in an ROC AUC score of 92.7% with the top 40 features from the non-overlapping dataset. Dominguez et al. validated their model on the DEAP dataset with an F1 score of 81% using bagging trees. Our DT algorithms performed better on both the overlapping and non-overlapping dataset—ROC AUC scores of 91.7 and 90%, respectively.

Fear was classified based only on the HRV features with an accuracy of 56.9% using the SVM algorithm [30]. SVM was also favored in [31], where the emotion of fear (considered as low valence and high arousal) was classified with an accuracy of 62.35% based on the physiological measurements from the DEAP dataset. Our method outperforms these studies by more than 25% for the SVM algorithm and more than 15% for the artificial neural network for the overlapping dataset.

The ENS resulted in an accuracy of 96.33% for the subject-dependent modality and 76.67% for the subject-independent modality using the Leave One Subject Out testing approach, based on the GSR, ECG and skin temperature signals of 12 subjects (only women) stored in the MAHNOB dataset [32]. Their classification results are close to ours, but a disadvantage stands in the fact that they relied on a lower number of subjects.

By collecting and using respiration rate and HRV in both time and frequency domains, fear classification accuracy using a CNN model reached 95.83%, but at the cost of high computational requirements [29].

Our methods for fear classification outperformed valence and arousal classification. Vij et al. [26] obtained a maximum F1 score for valence classification of 77.2%, and 77.5% for arousal classification using the SVM algorithm. Based on the data from DEAP, using PPG signals and the normal-to-normal intervals of HRV in the time domain and frequency domain, valence classification reached 82.1%, while arousal classification reached 80.9% using convolutional network configurations [19].

In our research from 2019 [34], the highest F1 scores were obtained by using the Random Forest Classifier with no feature selection, having as input the Higuchi Fractal Dimension of 32 EEG channels and the physiological recordings (hEOG, vEOG, zEMG, tEMG, GSR, Respiration, PPG and temperature)—89.96%; Random Forest Classifier and Fisher selection having as input the Approximate Entropy for each of the 32 EEG channels and the physiological recordings (hEOG, vEOG, zEMG, tEMG, GSR, Respiration, PPG and temperature)—89.51%; and Random Forest Classifier and Sequential Feature Selection using both EEG Power Spectral Densities and Higuchi Fractal Dimensions of the 32 EEG channels and the physiological recordings (hEOG, vEOG, zEMG, tEMG, GSR, Respiration, PPG and temperature)—81%. kNN resulted in the highest classification accuracy (87.45%) with the PCA feature selection and EEG Power Spectral Densities of the 32 channels and the physiological recordings (hEOG, vEOG, zEMG, tEMG, GSR, Respiration, PPG and temperature) as input.

In the current research, for the non-overlapping dataset, PCA dimensionality reduction and SVM led to an ROC AUC score of 93.5% and for the overlapping dataset, GBT reached an ROC AUC score of 91.7%. For the non-overlapping dataset, Recursive Feature Elimination for Feature Selection together with the SVM algorithm with the top 40 features resulted in an ROC AUC score of 92.7%. For the overlapping dataset, XGBoost Feature Selection or Random Forest Classification Feature Selection and kNN with the top 30 features reached an ROC AUC score of 82.5%. Thus, the current approach increases classification performance, relying only on the features extracted from the GSR and HR signals, after following a protocol which consists of denoising, segmentation, windowing, adjusting an imbalanced dataset and exhaustively searching for the most optimal classification models’ hyperparameters.

### 9.6. Limitations of the Current Study

One highly decisive aspect regarding the efforts made within the field of artificial intelligence and machine learning is the sustained attention given to the studies’ constraints. It is very important to have a meaningful interpretation of how each detail can change and impact the results of such experiments and whether the conclusions that one takes are universal or whether they are drawn from a certain framework.

In our situation, dealing with imbalanced classes represented one of the threshold problems. As is commonly the case, adjusting and refining the data we worked with implied creating our own boundaries.

SMOTE could be considered a limitation in this scenario. It was necessary in the evolution of our experiment due to its ability to balance the two classes; nevertheless, generating new data often comes with a cost. We performed data augmentation before feeding the algorithm with samples. Other studies relied on upsampling the minority class inside the cross-validation loop. Especially during the cross-validation trials, the means by which SMOTE can render new samples is disputable; the examples split between the validation and the training sets can be very similar. Thus, the classifier quickly recognizes those from the validation and test sets, and further provides a high, misleading accuracy that impacts real progress. However, we tried the inner upsampling as well, but the results varied little upon examination. We suspect that the neural networks suffer from overfitting as we judged and compared the results for cross-validation and test from Table 11 and Table 14, respectively. Thus, the cross-validation and test classification scores have an ascending trend, reaching very high values. The same observation can be made for the DT algorithms (Figure 6, Figure 7, Figure 12 and Figure 13).

Moreover, another tool was employed to further inspect the dependencies among data: single factor ANOVA, which tests whether the data are equal. The tests were performed on the standardized data that the networks used for training and testing. The tool confirmed that the samples were not susceptible to being identical or too similar (F > F critical, F = 20.45, F critical = 1.06).

In the DEAP database, participants performed a single self-assessment of the emotional status for the entire video extract. Because the emotional state of the participants may vary during the 60 s corresponding to the video extracts, a single evaluation may not correctly represent the evolution of the emotional states for each evaluation window. This issue can negatively affect the training process of the fear evaluation algorithms, leading to lower estimation accuracy.

## 10. Conclusions

In this paper, we performed a thorough analysis of the best methods for classifying the emotion of fear, considering various machine learning techniques. The results show that fear can be predicted by extracting the most relevant features from the electrodermal and heart rate data and by searching for the best parameters which maximize the algorithms’ classification scores. By performing various tweaks, such as resolving the problem of having an imbalanced dataset, reducing overfitting, testing various configurations, tuning the most important hyperparameters and identifying various technical and mathematical limitations, we reached a classification performance (measured with the ROC AUC score, which is the most reliable classification metric) that surpassed most of the studies in the literature or was comparable to some of the most recent ones (although few in number).

We have come to the conclusion that the classification scores are statistically equal, whether we use the algorithms with or without dimension reduction/feature selection. The differences in performance are small. However, we selected the algorithms which maximized the ROC AUC score—for the non-overlapping dataset, PCA dimensionality reduction + SVM—93.5% and for the overlapping dataset, GBT—91.7%. For the non-overlapping dataset, if we want to select fewer features and to preserve a high classification performance, the Recursive Feature Elimination for Feature Selection + SVM algorithm with the top 40 features had an ROC AUC score of 92.7%. For the overlapping dataset, XGBoost Feature Selection or Random Forest Classification Feature Selection + kNN with the top 30 features lead to an ROC AUC score of 82.5%.

The selection of features is consistent, as six of the top 10 features are common to all employed approaches and algorithms. Additionally, the six common features are relevant from the biological point of view, knowing from the literature that they are correlated with the activity of the ANS, which is involved in dealing with situations of stress and fear.

The deficiencies of the current study are detailed in Section 9.6. The main limitations lie in the fact that we worked on an imbalanced dataset that had to be artificially balanced using the SMOTE technique, the modality in which the signals were recorded—a single self-assessment of the emotional status for an entire video extract—and the particular data points which were missing or had flawed values in the dataset, affecting in this way the calculations and introducing errors in the features extracted.

The method developed in this study can have implications in both practice and research. For clinical applicability, we can mention the real-time monitoring of fear during anxiety treatment sessions, phobias and post-traumatic stress disorder. As a research target, this method can be used for developing and testing applications that require the real-time evaluation of the user’s emotional response, such as medical, educational, military, or sports training.

The technology application scenario involves a future development of a wearable device that uses these algorithms for clinical applicability in the treatment of anxiety by VR immersion therapy. Because the use of VR equipment does not allow an easy collection of EEG data or interpretation of facial expressions, we chose EDA and HR as the only viable options in these conditions.

As future directions of research, we also plan to apply the same feature extraction, classification and feature selection methods on data originating from other databases (such as MAHNOB [33]) and on data recorded from subjects who will play a virtual reality game dedicated to phobia therapy. We have developed and are currently developing various games for acrophobia [55], ophiophobia [56], claustrophobia [57], pyrophobia [58] and fear of public speaking therapy [59]. The best classification algorithms will be used to automatically estimate the in-game intensity of fear and to adjust the level of exposure to various threatening stimuli. Moreover, recent explainable methods can be employed in future work in order to provide a better understanding of the proposed model and achieved results, such as the EEG-based approach proposed for decoding open/closed hand motion preparation described in [60] and the brain-inspired spiking neural network architecture presented in [61].

## Figures and Tables

**Table 1:** Results of the SVM algorithm applied on the non-overlapping dataset.

	SVM	PCA Dimensionality Reduction + SVM	XGBoost Feature Selection + SVM	Pearson Feature Selection + SVM	L1 Regularization Feature Selection + SVM	Random Forest Classification Feature Selection + SVM	Recursive Feature Elimination Feature Selection + SVM
5-fold cross-validation training	86.7%	**92.7%**	-	-	-	-	-
5-fold cross-validation test	70.1%	**75.7%**	-	-	-	-	-
The best model	C = 10, gamma = 0.083 (1/n_features ∗ 10)	C = 1000, gamma = 0.083 (1/n_features ∗ 10)	110 features C = 10, gamma = 0.09 (1/n_features ∗ 10)	80 features C = 10, gamma = 0.125 (1/n_features ∗ 10)	90 features C = 10, gamma = 0.11 (1/n_features ∗ 10)	50 features C = 10, gamma = 0.2 (1/n_features ∗ 10)	40 features C = 1000, gamma = 0.25 (1/n_features ∗ 10)
Cross-validation grid search test	87.8%	**90.3**%	87.8%	89.1%	88.5%	87.7%	89.5%
F1 score	93%	**93.5**%	92.8%	92.8%	88.4%	92.8%	92.8%
ROC AUC score	92.7%	**93.5%**	92.1%	92.3%	87.7%	92.1%	92.7%
Accuracy	93%	**93.5**%	92.8%	92.8%	88.6%	92.8%	92.8%
Sensitivity	90%	**93.5**%	86%	88%	79.5%	85.5%	92%
Specificity	95.5%	93.5%	98.3%	96.7%	95.9%	**98.7**%	93.5%
Average F1 score (10 iterations)	91%	**92.3**%	-	-	-	-	-
Average ROC AUC score (10 iterations)	90.3%	**92**%	-	-	-	-	-
Average accuracy (10 iterations)	91%	**92.4**%	-	-	-	-	-
Average sensitivity (10 iterations)	83.9%	**89**%	-	-	-	-	-
Average specificity (10 iterations)	**96.8**%	95.1%	-	-	-	-	-

**Table 2:** Results of the DT algorithms applied on the non-overlapping dataset.

	GBT	RF
5-fold cross-validation training	**100%**	**100%**
5-fold cross-validation test	81%	**81.9%**
The best model	Iteration 9	max_depth = None, max_features = log2, min_samples_split = 10, n_estimators = 300
Cross-validation grid search test	-	**87.4%**
F1 score	**90.5%**	90.1%
ROC AUC score	**90%**	89.4%
Accuracy	**90.6%**	90.2%
Sensitivity	**84.5%**	83%
Specificity	95.5%	**95.9%**
Average F1 score (10 iterations)	**88.9%**	88.7%
Average ROC AUC score (10 iterations)	**88.4%**	88.2%
Average accuracy (10 iterations)	**89%**	88.8%
Average sensitivity (10 iterations)	**83.4%**	81.9%
Average specificity (10 iterations)	93.5%	**94.4%**

**Table 3:** Results of the kNN algorithm applied on the non-overlapping dataset.

	kNN	PCA Dimensionality Reduction + kNN	XGBoost Feature Selection + kNN	Pearson Feature Selection + kNN	L1 Regularization Feature Selection + kNN	Random Forest Classification Feature Selection + kNN
5-fold cross-validation training	**75.4%**	73.3%	-	-	-	-
5-fold cross-validation test	61.5%	**62.1%**	-	-	-	-
The best model	leaf_size = 5, n_neighbors = 4, *p* = 1	leaf_size = 42, n_neighbors = 4, *p* = 1	30 features leaf_size = 5, n_neighbors = 3, *p* = 1	90 features leaf_size = 5, n_neighbors = 4, *p* = 1	80 features leaf_size = 5, n_neighbors = 4, *p* = 1	60 features leaf_size = 5, n_neighbors = 4, *p* = 1
Cross-validation grid search test	75.8%	72.8%	**76.4%**	75.7%	75.4%	76%
F1 score	80.2%	78.6%	80.3%	**80.4%**	79.5%	80.2%
ROC AUC score	80.8%	79.6%	**81.4%**	81%	80.1%	80.5%
Accuracy	80.1%	78.6%	**80.4%**	**80.4%**	79.5%	80.1%
Sensitivity	87%	89%	**91%**	87%	86%	83.5%
Specificity	74.6%	70.2%	71.8%	75.1%	74.2%	**77.5%**
Average F1 score (10 iterations)	**77.2%**	75.1%	-	-	-	-
Average ROC AUC score (10 iterations)	**77.9%**	76.6%	-	-	-	-
Average accuracy (10 iterations)	**77.2%**	75.3%	-	-	-	-
Average sensitivity (10 iterations)	84.4%	**88.7%**	-	-	-	-
Average specificity (10 iterations)	**71.4%**	64.6%	-	-	-	-

**Table 4:** Top 10 features selected by the SVM and kNN algorithms for the non-overlapping dataset.

SVM	kNN
GSR_fmd[W3]	SCL_fmd[W2]
HRV_NN50[W3]	GSR_fmd[W1]
HRV_hlr[W1]	HRV_hlr[W1]
GSR_fmd[W2]	SCR_fmd[W1]
SCR_fmd[W1]	HRV_NN50[W3]
HRV_NN20[W2]	GSR_fmd[W3]
HRV_hlr[W3]	HRV_NN20[W2]
SCL_fmd[W1]	SCR_ssc[W2]
SCR_wamp[W1]	SCL_fmd[W3]
SCR_mav[W2]	SCL_fmd[W1]

**Table 5:** Results of the SVM algorithm applied on the overlapping dataset.

	SVM	PCA Dimensionality Reduction + SVM	XGBoost Feature Selection + SVM	Pearson Feature Selection + SVM	L1 Regularization Feature Selection + SVM	Random Forest Classification Feature Selection + SVM	Recursive Feature Elimination Feature Selection + SVM
5-fold cross-validation training	73.6%	**75.2%**	-	-	-	-	-
5-fold cross-validation test	66.2%	**67.2%**	-	-	-	-	-
The best model	C = 10, gamma = 0.25 (1/n_features ∗ 10)	C = 10, gamma = 0.25 (1/n_features ∗ 10)	40 features C = 10, gamma = 0.25 (1/n_features ∗ 10)	40 features C = 10, gamma = 0.25 (1/n_features ∗ 10)	40 features C = 10, gamma = 0.25 (1/n_features ∗ 10)	40 features C = 10, gamma = 0.25 (1/n_features ∗ 10)	40 features C = 10, gamma = 0.25 (1/n_features ∗ 10)
Cross-validation grid search test	85.3%	83.9%	85.1%	85.5%	85.5%	**86.2%**	86.1%
F1 score	88.9%	85.8%	**89.2%**	87.8%	88.1%	87%	87.5%
ROC AUC score	88.8%	86%	**89.2%**	87.8%	88%	86.9%	87.4%
Accuracy	88.9%	85.8%	89.2%	87.8%	88.1%	87%	87.5%
Sensitivity	87.4%	87.6%	**89.2%**	87.9%	86.7%	85.8%	86.8%
Specificity	**90.2%**	84.4%	89.1%	87.7%	89.3%	88%	88.1%
Average F1 score (10 iterations)	**87.9%**	86%	-	-	-	-	-
Average ROC AUC score (10 iterations)	**87.8%**	86.2%	-	-	-	-	-
Average accuracy (10 iterations)	**87.9%**	86%	-	-	-	-	-
Average sensitivity (10 iterations)	87%	**88.6%**	-	-	-	-	-
Average specificity (10 iterations)	**88.6%**	83.9%	-	-	-	-	-

**Table 6:** Results of the DT algorithms applied on the overlapping dataset.

	GBT	RF
5-fold cross-validation training	**100%**	**100%**
5-fold cross-validation test	**86.2%**	85.4%
The best model	Iteration 7	max_depth = None, max_features = sqrt, min_samples_split = 10, n_estimators = 300
Cross-validation grid search test	-	**89.3%**
F1 score	**92.2%**	90.2%
ROC AUC score	**91.7%**	89.6%
Accuracy	**92.2%**	90.3%
Sensitivity	**87.3%**	83.4%
Specificity	**96.2%**	95.8%
Average F1 score (10 iterations)	**91.7%**	89.8%
Average ROC AUC score (10 iterations)	**91.1%**	89.2%
Average accuracy (10 iterations)	**91.7%**	89.9%
Average sensitivity (10 iterations)	**85.4%**	82.2%
Average specificity (10 iterations)	**96.8%**	96.1%

**Table 7:** Results of the kNN algorithm applied on the overlapping dataset.

	kNN	PCA Dimensionality Reduction + kNN	XGBoost Feature Selection + kNN	Pearson Feature Selection + kNN	L1 Regularization Feature Selection + kNN	Random Forest Classification Feature Selection + kNN
5-fold cross-validation training	**81%**	80.6%	-	-	-	-
5-fold cross-validation test	**69.8%**	69.3%	-	-	-	-
The best model	leaf_size = 5, n_neighbors = 4, *p* = 1	leaf_size = 6, n_neighbors = 4, *p* = 1	30 features leaf_size = 5, n_neighbors = 3, *p* = 1	40 features leaf_size = 5, n_neighbors = 3, *p* = 1	40 features leaf_size = 5, n_neighbors = 4, *p* = 1	30 features leaf_size = 5, n_neighbors = 3, *p* = 1
Cross-validation grid search test	78.6%	75.7%	79.9%	80%	79.8%	80.6%
F1 score	84%	78.7%	81.7%	80.2%	81%	81.8%
ROC AUC score	**83.9%**	78.7%	82.5%	81.3%	81%	82.5%
Accuracy	**84%**	78.7%	81.7%	80.2%	81%	81.7%
Sensitivity	83.3%	79.3%	90%	**91.1%**	81%	89.3%
Specificity	**84.6%**	78.3%	75.1%	71.4%	81%	75.7%
Average F1 score (10 iterations)	**81.7%**	77.8%	-	-	-	-
Average ROC AUC score (10 iterations)	**81.8%**	78.2%	-	-	-	-
Average accuracy (10 iterations)	**81.7%**	77.8%	-	-	-	-
Average sensitivity (10 iterations)	**83.1%**	81.1%	-	-	-	-
Average specificity (10 iterations)	**80.6%**	75.2%	-	-	-	-

**Table 8:** Top 10 features selected by the SVM and kNN algorithms for the overlapping dataset.

SVM	kNN
SCL_fmd	SCL_fmd
SCR_fmd	GSR_fmd
HRV_NN20	SCR_fmd
GSR_fmd	HRV_NN50
HRV_NN50	HRV_hlr
HRV_hlr	HRV_NN20
GSR_mav	GSR_mav
SCL_mav	SCL_mav
HRV_pNN20	GSR_str
GSR_nimp	GSR_nimp

**Table 9:** Tested configurations for the non-overlapping dataset.

	Number of Hidden Layers	Number of Neurons on the Hidden Layer	Dropout	Number of Epochs
Config. 1	-	-	-	200
Config. 2	-	-	0.5 for the input layer	200
Config. 3	-	-	0.8 for the input layer	500
Config. 4	1	80	0.8 for the input layer	500
Config. 5	1	11	0.8 for the input layer	500

**Table 10:** Classification scores for Configurations 1–5, for the non-overlapping dataset.

	Config. 1	Config. 2	Config. 3	Config. 4	Config. 5
F1 score	87.5%	85.7%	86.3%	85.2%	83.7%
ROC AUC score	87.7%	85.5%	86.1%	85.9%	83.6%
Accuracy	87.5%	85.7%	86.4%	85.3%	83.7%
Sensitivity	90.2%	83.4%	82.5%	93.2%	82.5%
Specificity	85.1%	87.6%	89.7%	78.6%	84.7%

**Table 11:** Cross-validation and test scores for Configuration 3.

	Cross-Validation	Test (10 Runs Averaged)
F1 score	97.4%	92.8%
ROC AUC score	97.5%	92.4%
Accuracy	97.4%	92.8%
Sensitivity	98.4%	87.8%
Specificity	96.6%	97.1%

**Table 12:** Tested configurations for the overlapping dataset.

	Number of Hidden Layers	Number of Neurons on the Hidden Layers	Dropout	Number of Epochs
Config. 1	-	-	-	200
Config. 2	-	-	0.5 for the input layer	200
Config. 3	-	-	-	500
Config. 4	-	-	0.5 for the input layer	500
Config. 5	1	6	0.5 for the input layer	200
Config. 6	2	27 and 6	0.5 for the input layer	200
Config. 7	3	30, 20 and 10	0.5 for the input layer	200

**Table 13:** Classification scores for Configurations 1–7, for the overlapping dataset.

	Config. 1	Config. 2	Config. 3	Config. 4	Config. 5	Config. 6	Config. 7
F1 score	81%	77.6%	80.2%	77.2%	75.7%	73.7%	67.2%
ROC AUC score	80.8%	77.4%	80.6%	77.6%	76%	75.6%	71.3%
Accuracy	81%	77.5%	80.1%	77.1%	75.6%	73.9%	68.5%
Sensitivity	79.3%	76.9%	84.5%	81.4%	79.2%	90%	94.9%
Specificity	82.4%	78%	76.7%	73.8%	72.7%	61.3%	47.8%

**Table 14:** Cross-validation and test scores for Configuration 2.

	Cross-Validation	Test (10 Runs Averaged)
F1 score	85.7%	78.3%
ROC AUC score	85.6%	78.8%
Accuracy	85.8%	78.2%
Sensitivity	83.8%	83.9%
Specificity	87.3%	73.7%

**Table 15:** *p* and F values for ANOVA test for independent measures, non-overlapping dataset.

	SVM		DT		kNN	
	Grid search for the best configuration	Average over 10 iterations	Grid search for the best configuration	Average over 10 iterations	Grid search for the best configuration	Average over 10 iterations
*p*	0.48	0.51	0.85	0.92	0.98	0.72
F	0.92	0.47	0.03	0.008	0.13	0.12

**Table 16:** *p* and F values for SVM, DT and kNN algorithms, overlapping dataset.

	SVM		DT		kNN	
	Grid search for the best configuration	Average over 10 iterations	Grid search for the best configuration	Average over 10 iterations	Grid search for the best configuration	Average over 10 iterations
*p*	0.012	0.06	0.41	0.52	0.37	0.006
F	3.93	4.65	0.72	0.44	1.1	13.64

## Data Availability

Not applicable.
